# Carbon Monoxide Interacts with Auxin and Nitric Oxide to Cope with Iron Deficiency in *Arabidopsis*

**DOI:** 10.3389/fpls.2016.00112

**Published:** 2016-03-07

**Authors:** Liming Yang, Jianhui Ji, Hongliang Wang, Karen R. Harris-Shultz, Elsayed F. Abd_Allah, Yuming Luo, Yanlong Guan, Xiangyang Hu

**Affiliations:** ^1^Jiangsu Collaborative Innovation Center of Regional Modern Agriculture and Environment Protection, Jiangsu Key Laboratory for Eco-Agriculture Biotechnology around Hongze Lake, Huaiyin Normal UniversityHuaian, China; ^2^Crop Protection and Management Research Unit, Agricultural Research Service – United States Department of AgricultureTifton, GA, USA; ^3^Department of Plant Pathology, The University of GeorgiaTifton, GA, USA; ^4^Crop Genetics and Breeding Research Unit, Agricultural Research Service – United States Department of AgricultureTifton, GA, USA; ^5^Department of Plant Production, College of Food and Agricultural Sciences, King Saud UniversityRiyadh, Saudi Arabia; ^6^Key Laboratory of Biodiversity and Biogeography, Kunming Institute of Botany, Institute of Tibet Plateau Research at Kunming, Chinese Academy of SciencesKunming, China

**Keywords:** CO, NO, auxin, iron deficiency, *Arabidopsis*, basipetal transport

## Abstract

To clarify the roles of carbon monoxide (CO), nitric oxide (NO), and auxin in the plant response to iron deficiency (–Fe), and to establish how the signaling molecules interact to enhance Fe acquisition, we conducted physiological, genetic, and molecular analyses that compared the responses of various *Arabidopsis* mutants, including *hy1* (CO deficient), *noa1* (NO deficient), *nia1/nia2* (NO deficient), *yuc1* (auxin over-accumulation), and *cue1* (NO over-accumulation) to –Fe stress. We also generated a HY1 over-expression line (named HY1-OX) in which CO is over-produced compared to wild-type. We found that the suppression of CO and NO generation using various inhibitors enhanced the sensitivity of wild-type plants to Fe depletion. Similarly, the *hy1*, *noa1*, and *nia1*/*nia2* mutants were more sensitive to Fe deficiency. By contrast, the *yuc1*, *cue1*, and HY1-OX lines were less sensitive to Fe depletion. The *hy1* mutant with low CO content exhibited no induced expression of the Fe uptake-related genes *FIT1* and *FRO2* as compared to wild-type plants. On the other hand, the treatments of exogenous CO and NO enhanced Fe uptake. Likewise, *cue1* and HY1-OX lines with increased endogenous content of NO and CO, respectively, also exhibited enhanced Fe uptake and increased expression of bHLH transcriptional factor FIT1as compared to wild-type plants. Furthermore, we found that CO affected auxin accumulation and transport in the root tip by altering the PIN1 and PIN2 proteins distribution that control lateral root structure under –Fe stress. Our results demonstrated the integration of CO, NO, and auxin signaling to cope with Fe deficiency in *Arabidopsis*.

## Introduction

Iron deficiency (–Fe) severely limits the yield of crops growing in calcareous soils (Curie and Briat, 2003). Although Fe is abundant in the soil, Fe availability is limited due to its poor solubility in aerobic and neutral pH environments (Briat et al., 1995). Plants have evolved two distinct strategies of Fe acquisition to cope with –Fe (Imsande, 1998). Non-graminaceous plants acidify the extracellular medium surrounding the roots and improve the ferric-reducing capability of the roots to enhance ferrous Fe uptake (Strategy I), whereas graminaceous plants secrete phytosiderophores to enhance Fe uptake from the soil (Strategy II) (Marschner, 2011). In *Arabidopsis* (*Arabidopsis thaliana*), ferric Fe reduction is mainly achieved through the activity of Fe reductase, FRO2 (Ferric Reductase Oxidase 2), on the surface of roots. Ferrous Fe is then absorbed through the root epidermis by the metal transporter IRT1 (Iron-Regulated Transporter 1) (Vert et al., 2002). Fe-Deficiency Induced Transcription Factor 1 (FIT1) forms heterodimers with basic Helix-Loop-Helix (bHLH) 38 and bHLH39 to regulate Fe acquisition (Yuan et al., 2008). Furthermore, two other bHLH proteins, bHLH 100 and bHLH 101, are highly induced by –Fe, which indicates their involvement in Fe acquisition (Wang et al., 2007; Sivitz et al., 2012). The intercellular distribution of Fe throughout the plant also affects Fe availability. Ferric Reductase Defective 3 (FRD3), an eﬄux transporter of ion chelator citrate, facilitates root-to-shoot circulation of iron in the xylem sap (Green and Rogers, 2004). Nicotianamine (NA) is a low molecular mass metal chelator with a high binding affinity for a range of transition elements including Fe. The Fe transporter Yellow Stripe-like 1 (YSL1) moves Fe (II)-NA from the xylem to the phloem (Le Jean et al., 2005). Various signaling molecules and plant hormones including nitric oxide (NO), auxin and ethylene modulate the response to –Fe by plants (Graziano and Lamattina, 2007; Chen et al., 2010; Lingam et al., 2011; Meiser et al., 2011). In addition, Fe uptake is regulated at the post-transcriptional level. For instance, FIT and IRT undergo ubiquitin-dependent protein degradation in relation to Fe availability (Barberon et al., 2011; Meiser et al., 2011). However, many of the mechanisms underlying Fe acquisition during plant exposure to –Fe remain to be identified and characterized.

Carbon monoxide (CO), an important reactive trace gas in the troposphere, has recently been shown to be generated in animals through the activity of heme oxygenase (HY; EC1.14.99.3), and is an essential regulator (Otterbein et al., 2000). Recent research in plants suggests that CO plays a role in root formation, and in the response to saline and heavy metal stress (Guo et al., 2008; Han et al., 2008; Xuan et al., 2008; Xie et al., 2011). Likewise, NO is a free radical gas involved in the regulation of multiple physiological functions in animals and plants (Wendehenne et al., 2004; Yang et al., 2013, 2015). In plants, NO is generated through the activities of nitrate reductase (NR) and NO synthase (NOS)-like enzymes, and possibly also non-enzymatic processes (Besson-Bard et al., 2008; Scheler et al., 2013). Under chilling stress, NO normally interacts with CO to regulate seed germination (Bai et al., 2012). In *Arabidopsis*, long-hypocotyl protein 1 (HY1), a heme oxygenase, has been shown to participate in the biosynthesis of the phytochrome chromophore (Davis et al., 1999). Recently the role of HY1 in CO synthesis has drawn much attention (Han et al., 2008; Xuan et al., 2008; Xie et al., 2011). A previous study suggests that HY1-dependent CO generation induces the subsequent accumulation of NO, thereby improving the tolerance of *Arabidopsis* to –Fe (Li et al., 2013). Auxin also induces NO accumulation which increases the activity of ferric chelate reductase (FCR) to promote Fe uptake in *Arabidopsis* subjected to –Fe (Chen et al., 2010). It has been suggested that there are possible interplays among CO, NO, and auxin signaling upon plant exposure to –Fe. However, the patterns of these signaling interactions in response to iron depletion remains to be characterized further.

## Materials and Methods

### Plant Growth and Treatments

The Columbia ecotype (Col-0) of *Arabidopsis* served as wild-type and all mutants in the Col-0 background were obtained from the *Arabidopsis* stock center (Scholl et al., 2000), including the HY1 null mutant *hy1*, the auxin-insensitive mutants *axr1-3* (Lincoln et al., 1990), the auxin-transport mutant *aux1-7* (Pickett et al., 1990), the auxin-overproducing mutant *yuc1* (Zhao et al., 2001), the NO overproducing mutant *cue1* (He et al., 2004), the NOA-deficient mutant *noa1* (Guo and Crawford, 2005), the NR-null-deficient double mutant *nr (nia1/nia2)*, the auxin-related reporter lines *DR5:GFP* (Ottenschläger et al., 2003)*, AUX1-YFP* (Giehl et al., 2012), *PIN1-GFP* (Ottenschläger et al., 2003), and *PIN2-GFP* (Ottenschläger et al., 2003). The double mutants, *yuc1/hy1* and *hy1/cue1*, were generated by crossing. The resulting homozygous lines were identified and isolated using the PCR primers listed in **Supplementary Table S1**.

Seeds were surface sterilized, kept in dark at 4°C for three days, and then germinated on 0.9% (w/v) agar plates supplemented with 1.0% (w/v) sucrose and a nutrient solution, consisting of 300 μM Ca(NO_3_)_2_, 50 μM MgSO_4_, 30 μM NaH_2_PO_4_, 50 μM K_2_SO_4_, 3 μM H_3_BO_3_, 0.4 μM ZnSO_4_, 0.2 μM CuSO_4_, 0.5 μM MnCl_2_, 1 μM (NH4)_6_(Mo_7_)O_24_, and 20 μM Fe-EDTA at pH 6.5 (KOH). One-week old seedlings were transferred into vermiculite supplemented with the same nutrient solution as described above. After another week, the seedlings were transplanted from vermiculite into the compartments of 1 L hydroponic holders filled with the nutrient solution described above with aeration. The nutrient solution was refreshed every other day. The seedlings were then grown at 21°C with a relative humidity of 70% and a daily light-dark cycle of 10 and 14 h. The daytime light intensity was 200 to 250 μmol photons m^-2^s^-1^. One week later, plants were transferred into the media containing either 20 μM (+Fe) or 0 μM (–Fe) FeNaEDTA for another week. The media was refreshed daily.

### Generation of Transgenic HY1-6HA-Overexpressing Plants

The cDNA of the *HY1* gene was synthesized through reverse transcription and served as the template to PCR amplify *HY1* fragments using the primers listed in **Supplementary Table S1**. The resulting HYI fragment was cloned into a pMD18 T-vector. The *HY1* insert was then excised using *EcoR*I and *BamH*I, and cloned into the *EcoR*I and *BamH*I sites of the *pOE-6HA* vector to obtain the expression vector *pOE-HY1-6HA*, in which the coding region of *HY1* was fused with 5′ end of a 6XHA (hemagglutinin) reporter tag. *pOE-HY1-6HA* was then transformed into Col-0 plants using *Agrobacterium tumefaciens* strain GV3101. Transgenic seeds were screened based on Basta resistance and the homozygous transgenic lines, named *HY1*-OX, were further verified by PCR and immunoblot analysis using anti-HA antibodies.

### Chemical/Inhibitor Treatments

The final concentrations of chemicals added to the culture solutions were as follows: 100 nM NAA, 10 μM NPA, 30 μM GSNO, 30 μM SNAP, 100 nM CORM2, 1 mM L-NAME, 10 mM tungstate, 300 μM cPTIO, and 200 μM ZnPPIX. Prior to inhibitor treatment, all lines were initially grown in +Fe media for 2 weeks. Afterward, all lines were transferred to –Fe media or –Fe media supplemented with various inhibitors and cultured for 1 week. Lines transferred to new +Fe media served as controls. After the indicated periods of treatments, the seedlings were collected for further analysis.

### CO Quantification and Assay of HY1 Enzyme Activity

Carbon monoxide in plant tissues was quantified using a previously described method with minor modifications (Bai et al., 2012). Briefly using liquid nitrogen, 0.5 g of treated plants were ground into a fine powder and transferred into a 4 ml bottle. Samples were stored under vacuum conditions in an ultralow chiller prior to further processing. To avoid foaming, 20 μl 1-octanol and 1 ml distilled water were sequentially added to each bottle. Each bottle was then immediately capped and shaken vigorously for approximately 30 s. One milliliter of sulfuric acid was injected into each bottle through the rubber cap using a syringe with a needle. Bottles were shaken briefly, and then placed into a 70°C water bath for 3 h with occasional shakings. After each bottle was cooled down to room temperature, 1 ml of air from the headspace was taken to quantify the CO concentration using a GC/MS system. The HY1 enzyme activity was determined as previously described (Bai et al., 2012).

### NO *In situ* Measurement

Nitric oxide content in root was quantified using DAF-FMDA under epifluorescence microscopy (Guo et al., 2003; Chen et al., 2010). Five millimeter of root tip segments were soaked in 20 mM HEPES/NaOH buffer (pH 7.4) supplemented with 5 mM DAF-FMDA for 20 min. After washing three times with 20 mM HEPES/NaOH buffer, the root tips were analyzed microscopically (Nikon Eclipse 80i, Nikon, EX 460-500, DM 505, BA 510-560). The intensities of the green fluorescence from the root tips were quantified by measuring the average pixel intensity with Photoshop software (Adobe Systems) (Guo et al., 2003). Data are presented as the mean percentages of fluorescence intensity relative to that of the wild-type plants grown under the same conditions.

### Measurement of Fe Content

Seedlings were washed for 5 min in a solution containing 5 mM CaSO_4_ and 10 mM EDTA, and rinsed briefly in de-ionized water prior to further processing. Roots and shoots were cut into smaller pieces, and dehydrated at 70°C. A hundred milligrams of the dried tissues were digested completely in 70% HNO_3_ at 120°C. The Fe content was determined using an Inductively Coupled Plasma-Optical Emission Spectrometer (ICP–OES, Perkin Elmer Optima 2100DV).

### Immunoblot Analysis

Total proteins of roots were prepared by grinding on ice with an extraction buffer consisting of 50 mM Tris, 5% glycerol, 4% SDS, 1% polyvinylpolypyrrolidone, and 1 mM phenylmethylsulfonyl fluoride (pH 8.0), followed by 14,000 × *g* centrifugation at 4°C for 15 min. Fifteen milligrams of total protein were separated by electrophoresis on a 12% SDS–polyacrylamide gel and blotted onto polyvinylidene difluoride membranes, which were then probed with the appropriate primary anti-HA antibody (1:1000) and horseradish peroxidase-conjugated goat anti-mouse secondary antibody (1:3000, Promega). Protein levels were visualized using an ECL Kit (GE healthcare, USA).

### Assay of Auxin Polar Basipetal Transport

Auxin transport was assayed using [^3^H] IAA as described previously (Guo et al., 2003). Briefly, the primary roots of 12-days-old seedlings were removed from intact plants. Then the seedlings, without primary roots, were placed onto petri dishes with 0.8% agar supplemented with half-strength Murashige and Skoog medium and additional chemicals for various treatments. For the assay of auxin polar basipetal transport, ten 1 cm hypocotyl segments excised below the cotyledons were carefully transferred into a microcentrifuge tube containing 0.1 mL of [^3^H]IAA (American Radiolabeled Chemicals) in an orientation of apical end down. After incubation for various periods, 0.2 cm segments from the apical end were excised and used to measure radioactivity in a liquid scintillation counter. The non-polar transport of auxin in the segments was determined by adding NPA (Chem Service) to the medium or by reversing the orientation of the stem segments in the medium.

### Determination of FCR Activity in Roots

Ferric Chelate Reductase activity was determined according to a method reported previously (Chen et al., 2010). Briefly, 0.1 g of the whole roots excised from seedlings were transferred into a test tube filled with 5 mL of assay solution consisting of 0.5 mM CaSO_4_, 0.1 mM 4-morpho-lineethanesulfonic acid, 0.1 mM bathophenanthrolinedisulfonic acid disodium salt hydrate (BPDS), and 100 mM Fe-EDTA (pH 5.5 using NaOH). The tubes were kept in the dark at room temperature for 1 h with brief swirling by hand every 10 min. The absorbance of samples at 535 nm was measured using a spectrophotometer, and the concentration of Fe(II)[BPDS]_3_ was calculated using an extinction coefficient of 22.14 mM^-2⋅^cm^-2^. Data were expressed as the mean percentages of FCR activity of the wild-type control grown on +Fe medium. To localize the distribution of FCR activities in roots, roots excised from the seedlings were embedded in 0.75% agarose medium containing 0.5 mM CaSO_4_, 0.5 mM FeNaEDTA, and 0.5 mM ferrozine. Roots were then incubated at room temperature for 20 min. Color patterns of 5 mm root tips were imaged by light microscopy (Nikon Eclipse 80i, Nikon).

### RNA Extraction and qRT-PCR

Total RNA was extracted from *Arabidopsis* seedlings using Trizol reagent (Invitrogen, USA). qRT-PCR was performed as described previously (Chen et al., 2010). Briefly, 1.5 μg of DNA-free RNA served as the template to synthesize first-strand cDNA in a 20 μL reaction using a Thermo Scientific RevertAid First Strand cDNA Synthesis Kit (Thermo Scientific, USA) and oligo (dT)18 primer. qRT-PCR was performed using 2x SYBR Green I Master Mix on a Roche Light Cycler 480 real-time PCR machine, according to the manufacturer’s instructions. At least three biological replicates were analyzed. *ACTIN2* served as an internal control of gene expression. Gene-specific primers that were used are listed in **Supplementary Table S1**.

### GUS Staining

The *HY1* promoter was cloned into the *pSUNG* vector using the primers listed in **Supplementary Table S1**. The resulting *pHY1:GUS* vector was transformed into Col-0 using the floral dip method (Clough and Bent, 1998). Basta-resistant T2 seedlings was used in the GUS staining assay as described previously (Bai et al., 2012).

### Histochemical Analysis

The activities of FCR and chlorophyll content were measured as described previously (Long et al., 2010). To localize Fe^3+^, 2-week-old seedlings were vacuum infiltrated with Perl’s stain solution containing 2% HCI and 2% K-ferrocyanide for 30 min. Seedlings were then incubated for another 30 min in Perl’s staining solution. After washing three times with distilled water, seedlings were imaged using a Leica DM500B microscope.

### Other Methods

Root growth rates were measured by marking the position of the root tips daily. Two-week-old seedlings were scanned and ImageJ software was used to measure root length. Lateral roots and lateral root primordia were counted under light microscopy.

Rhizosphere acidification was performed as described previously (Long et al., 2010). Briefly, seeds were germinated in +Fe medium and then were transferred into –Fe medium and grown for 1 week. The seedlings were then transferred onto a 1% agar plate (pH 6.5 using NaOH) containing 0.006% Bromocresol Purple and 0.2 mM CaSO_4_ and cultured for 24 h.

## Results

### Fe Depletion Induces Heme Oxygenase Activity and CO Production

**FIGURE 1:**
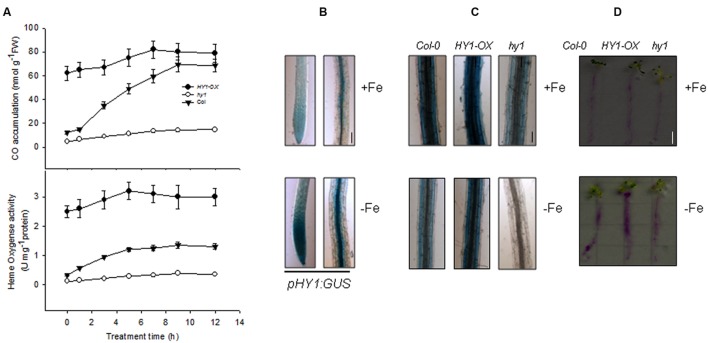
**Induction of heme oxygenase activity and CO generation by –Fe. (A)** –Fe induced heme oxygenase activity and CO generation. Two-week-old wild-type Col-0, *hy1* seedlings and HY1-OX lines were transferred to –Fe hydroponic medium, and heme oxygenase activity and CO generation were measured at the indicated time points. Data are the means ± SD (*n* = 6). **(B)** –Fe induced the transcription of *HY1*. Two-week-old wild-type Col-0 or transgenic *pHY1:GUS* seedlings were shifted to –Fe hydroponic medium, and the seedlings were subjected to GUS staining after 3 days of –Fe stress. Bar = 0.1 cm. **(C,D)** Perl staining analysis of Col-0, *hy1*, and 2-week-old *HY1-OX* plants subjected to –Fe stress. Two-week-old Col-0, *hy1*, and *HY1-OX* seedlings were shifted to –Fe liquid media for 1 week and the roots were analyzed using a ferrozine assay©, or to –Fe solid media containing Bromocresol Purple **(D)** for 1 day. Red indicates acidification. Bar = 0.5 cm.

To understand the regulatory role of CO in *Arabidopsis* plants exposed to –Fe, we first obtained *HY1*-null lines *hy1-100* (hereafter *hy1*), which harbors a single base pair mutation in the second exon of the *HY1* gene encoding heme oxygenase. The *hy1* mutant had yellowish leaves and longer hypocotyls as compared to wild-type Columbia after 5 days of continuous white light (Davis et al., 1999). We also generated several independent transgenic lines in which *HY1-6HA* was constitutively expressed under the control of CaMV 35S promoter. Among them, *HY1-6HA* expression was up-regulated most strongly in the line 3 and was named *HY1*-*OX* (**Supplementary Figure S1**). Thus, this line was used in the following experiments. First, we measured the heme oxygenase activity and CO release after Fe depletion for wild-type, *hy1*, and *HY1*-*OX* plants. As shown in **Figure 1A**, –Fe gradually induced heme oxygenase activity and CO production in the wild-type as well as increased *HY1* transcript abundance (**Supplementary Figure S2**). Compared to the wild-type, the *hy1* mutant, impaired in heme oxygenase activity, produced less CO. We also generated a transgenic line, *pHY1:GUS*, in which the promoter region of *HY1* drives the expression of the beta-glucuronidase (GUS) reporter gene. Consistent with the findings described above, the *pHY1:GUS* reporter line showed enhanced GUS staining under Fe depletion compared to wild-type (**Figure 1B**), indicating *HY1* expression and enzyme activity is inducible by Fe deficiency.

To determine the role of CO in regulating Fe uptake, we measured Fe content using two distinct techniques. Perl staining revealed that the wild-type and *HY1*-OX line contained more ferric Fe in their roots compared to the *hy1* line defective in the activity of heme oxygenase under Fe depletion (**Figure 1C**). The abundance of ferric Fe was also reduced in the wild-type and *HY1*-OX under –Fe, but ferric Fe was at such a low amount in the *hy1* line that it was not detectable. Similarly, the acidification capability of wild-type and *HY1*-OX was markedly higher than the *hy1* line under Fe depletion (**Figure 1D**). This suggests that HY1-dependent CO production plays a pivotal role in Fe uptake under Fe deficiency.

### Carbon Monoxide Enhances the Activity of FCR and Increases Fe Uptake in *Arabidopsis* Exposed to Fe Deficiency

**FIGURE 2:**
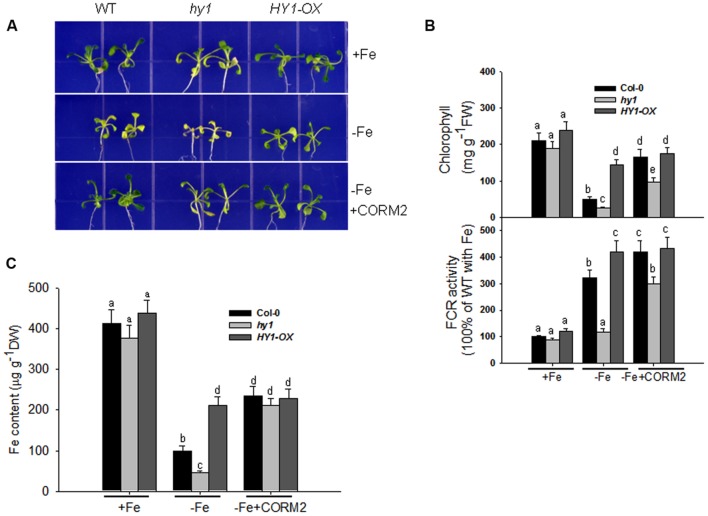
**The differential responses of the *hy1* mutant and *HY1-OX* line to Fe deficiency. (A)** The phenotype of wild-type Col-0, *hy1*, and *HY1-OX* lines in response to –Fe stress. Upper: +Fe media; Middle panel: –Fe media; Bottom: –Fe media plus the CO donor, CORM2. Col-0, *hy1*, and *HY1-OX* seeds were germinated for 7 days and then grown on vermiculite with fresh complete nutrient solution for another 7 days. The seeds were transferred to –Fe hydroponic media or –Fe hydroponic media plus 100 nM CORM2 for 1 week and then photographed. Seedlings growing in the +Fe hydroponic media were used as the positive control. **(B,C)** Quantification of chlorophyll content **(B)**, FCR activity **(B)**, and Fe content **(C)** of plants growing in +Fe or –Fe hydroponic media. Two-week-old seedlings of the Col-0, *hy1*, and *HY1-OX* lines were grown in +Fe hydroponic media, were shifted to –Fe hydroponic media for 1 week, and the chlorophyll content and FCR activity were measured. Seedlings left in +Fe hydroponic media were used as the control. Data are the means ± SD (*n* = 12), and bars labeled with different letters are significantly different at *p* < 0.05 (Tukey’s test).

We recorded the visible phenotypes of wild-type, *hy1*, and *HY1*-OX under Fe depletion. The wild-type and *HY1*-OX line grew well under regular conditions, but *hy1* was chlorotic (**Figure 2A**). Upon exposure to –Fe, the chlorophyll content and Fe content were both reduced in all lines as compared to +Fe (**Figures 2B,C**). However, the reductions were less in wild-type and *HY1*-OX plants as compared to *hy1* (**Figures 2B,C**). FCR activity was enhanced upon exposure to –Fe stress in the wild-type and *HY1*-OX plants but not in the *hy1* mutant. To determine whether CO is involved in the response to –Fe, we added the exogenous CO donor CORM2 to Fe-depleted medium. As shown in **Figure 2B**, the addition of CORM2 improved chlorophyll content and FCR activity in the wild-type and *hy1* mutant. Furthermore, the addition of exogenous CO under –Fe conditions alone significantly improved Fe content in the wild-type and *hy1* lines as compared to –Fe treatment (**Figure 2C**).

### Auxin Induces *HY1* Expression

**FIGURE 3:**
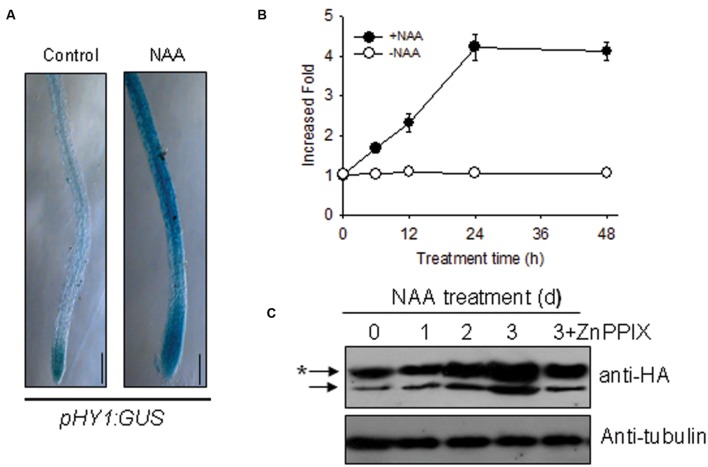
**Auxin induced *HY1* activity and *HY1*-dependent CO generation. (A)** Auxin treatment induced GUS expression in the *pHY1: GUS* transgenic line. Two-week-old *pHY1: GUS* seedlings were treated with 100 nM NAA for 1 day, and HY1 expression was monitored by GUS staining. Seedlings not exposed to NAA treatment were used as a control. Bar = 0.2 cm. **(B)** Time-course qRT-PCR analysis of *HY1* transcripts abundance after treatment with 100 nM NAA. **(C)** Auxin induced HY1-HA protein accumulation. Transgenic *HY1-OX* seedlings (termed *HY1-OX)* were treated with 100 nM NAA at the indicated time points, and HY1-HA accumulation was analyzed by immunoblot using anti-HA antibody. Blotting with an anti-tubulin antibody was used as a loading control. 3d+ZnPPIX: *HY1-OX* lines treated with 100 nM NAA plus 200 μM ZnPPIX for 3 days, before GFP-HY1 accumulation was measured. The asterisk indicates putative post-translational modification bands.

Because auxin signaling is involved in the *Arabidopsis* response to –Fe (Chen et al., 2010), we deduced that auxin and CO signaling processes may interact somehow in response to –Fe. To this end, we examined the effect of auxin on *HY1* expression. As shown in **Figure 3A**, the treatment of low concentration of NAA enhanced GUS staining in transgenic *pHY1:GUS* lines. Consistent with this observation, NAA treatment increased the abundance of *HY1* transcripts (**Figure 3B**). We also measured the impact of auxin on CO production. As shown in **Supplementary Figure S3**, NAA treatment induced CO generation and activity of heme oxygenase. In the *HY1*-OX line, NAA treatment enhanced HY1 protein accumulation, and the effect was reduced if HY1-OX was treated with ZnPPIX, a specific inhibitor of HY1 (**Figure 3C**). Interestingly, two protein fragments were identified in the samples at all timepoints, suggesting that possible post-translational modification of *HY1* occurs. These findings indicate the possible post-translational modification of HY1 after auxin treatment.

### CO Modulates Auxin, the Expression of the Auxin Transport Components, and Root Hair Development Under Fe Deficiency

**FIGURE 4:**
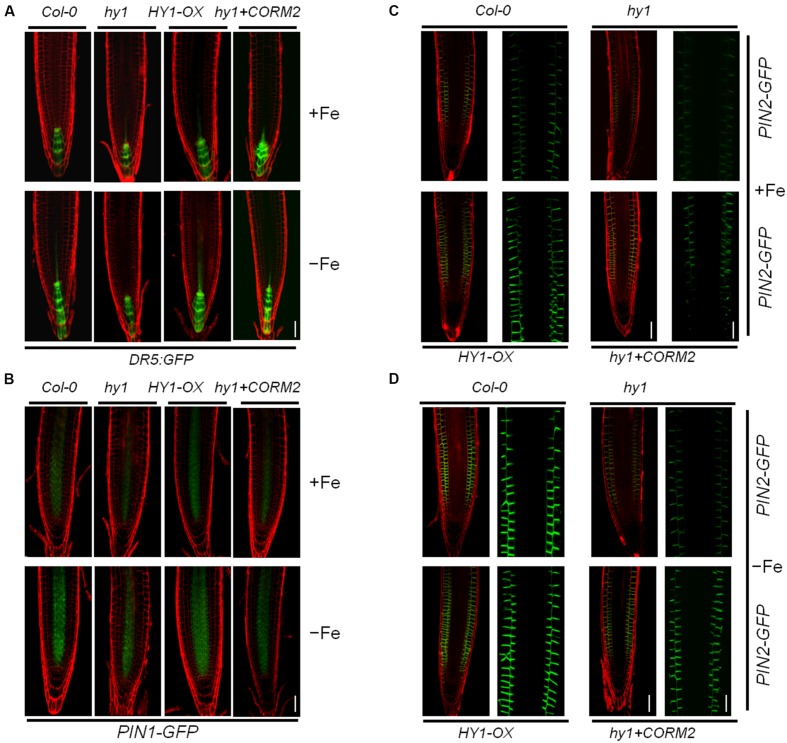
**Iron deficiency stress altered the expression patterns of *Dr5: GFP*, *PIN1-GFP*, and *PIN2-GFP* in the root tip. (A,B)** Confocal images of the *DR5:GFP* reporter **(A)** and *PIN1-GFP* **(B)** in Col-0, *hy1*, and *HY1-OX* lines treated with +Fe, –Fe, or –Fe +100 nM CORM2 for 1 day. **(C,D)** Confocal images of the *PIN2-GFP* reporter in Col-0, *hy1*, and *HY1-OX* lines treated with +Fe, –Fe, or –Fe +100 nM CORM2 for 3 days. Left: fluorescence images of the root tip; Right: enlarged window.

To gain insight into the role of CO in auxin signaling, we investigated the distribution of auxin in *hy1* and *HY1*-OX lines in response to the change of CO levels. We used an *Arabidopsis DR5:GFP* line in which DR5, a synthetic auxin-inducible promoter, drives the expression of the GFP reporter gene (Ulmasov et al., 1997). The line is widely used to detect the accumulation and distribution of auxin (Ottenschläger et al., 2003; Müller and Sheen, 2008). We transferred the *DR5:GFP* cassette into *hy1* and *HY1*-OX lines through genetic crossing. The analysis of confocal microscopy revealed that the GFP signal was stronger in the wild-type and *HY1*-OX lines than in the *hy1* mutant grown on +Fe media, and that the difference was enhanced further under Fe depletion (**Figure 4A**). Moreover, the CO donor CORM-2 treatment restored the auxin level in the root tip of *hy1* to that of the wild-type (**Figure 4A**). This may suggest that HY1-dependent CO production is positively correlated with the abundance of auxin in root tips.

The flux of auxin from the shoot to the root is largely determined by the polar localization of auxin transporters, including the auxin influx carrier AUX1 and eﬄux carriers PIN1 and PIN2, on the plasma membrane (Blilou et al., 2005). To investigate the polar localization of AUX1 and PIN1/2 in wild-type, *hy1*, and *HY1*-OX, *AUX1-YFP*, *PIN1-GFP*, and *PIN2-GFP* constructs were introgressed into these lines by crossings. We found that Fe depletion for one day dramatically reduced the abundance of *PIN2-GFP*, and to a lesser extent, of *PIN1-GFP* and *AUX1-YFP*, in *hy1* mutant compared with wild-type (**Figures 4B,C**; **Supplementary Figure S4**).

Iron deficiency also increased the root hair density in the wild-type and HY1-OX lines (**Supplementary Figure S5A,B**). The *hy1* mutant impaired in CO generation did not have an increase in root hair density induced by –Fe yet the addition of exogenous CO increased root hair formation (**Supplementary Figure S5A,B**). A similar observation has been reported previously (Graziano and Lamattina, 2007). The CO signal was also involved in lateral root development (**Supplementary Figure S5C**). The *HY1*-OX line developed more lateral roots under +Fe or –Fe conditions, and the *hy1* mutant had fewer lateral roots under –Fe conditions compared to the wild-type. –Fe treatment caused an increase in lateral root number in wild-type and *HY1*-OX lines, but not in *hy1*, which had fewer lateral roots even under +Fe conditions compared to wild-type (**Supplementary Figure S5C**). Addition of CORM2 under –Fe caused an increase in the root length of *hy1* (**Supplementary Figure S5C**).

### CO Increases FCR Activity, Fe Content, and Polar Auxin Transport but Requires Auxin Transporters

**FIGURE 5:**
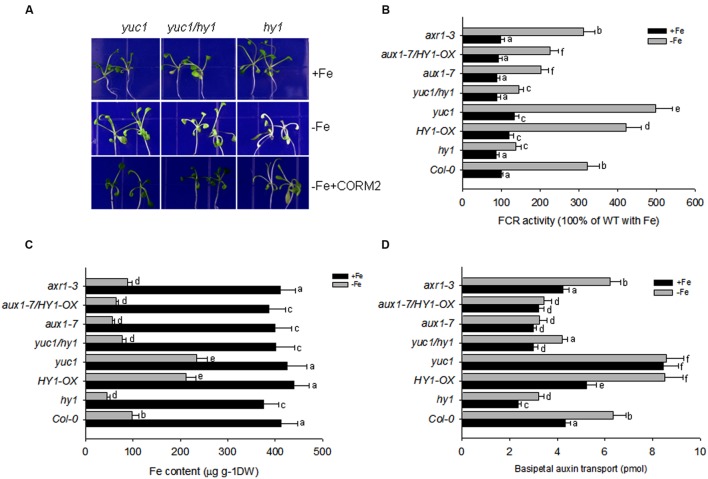
**Auxin and CO modulate the response to –Fe in *Arabidopsis*. (A)** The phenotypes of *yuc1*, *yuc1/hy1*, and *hy1* seedlings grown on +Fe, –Fe, or –Fe+CORM2 media for 1 week. These lines were all first grown on +Fe media for 2 weeks. Lines kept on +Fe media were used as the positive control. **(B–D)** The effect of –Fe stress on FCR activity **(B)**, Fe content **(C)**, and basipetal auxin transport **(D)** in the wild-type Col-0, *hy1*, and *HY1-OX* lines. All lines were first grown on +Fe media for 2 weeks and then shifted to –Fe media (–Fe) or kept on +Fe media (+Fe) for 1 week before measurements were taken. Data are the means ± SD (*n* = 10), and bars with different letters are significantly different at *p* < 0.05 (Tukey’s test).

To further characterize the modulation of auxin in response to –Fe, we crossed the auxin over-accumulation mutant *yuc1* with *hy1* to obtain the double mutant *yuc1/hy1* (Chen et al., 2010). Compared with *hy1*, the *yuc1* mutant was less chlorotic under –Fe, indicating an increased tolerance to –Fe (**Figure 5A**). Compared to wild-type, *yuc1* had increased iron content, FCR activity, and basipetal auxin transport after –Fe treatment for 1 week (**Figure 5B**). The addition of CORM-2 improved the growth of both the *hy1* and *yuc1/hy1* (**Figure 5A**). *HY1*-OX and *yuc1* had higher root Fe content and higher FCR activity after exposure to –Fe stress compared to wild-type (**Figures 5B,C**). Like *hy1*, *yuc1/hy1* double mutant had lower Fe content and FCR activity as compared to the wild-type plants (**Figures 5B,C**), implying that HY1 activity is essential/required for Fe acquisition during –Fe.

Because CO serves as a signal molecule to modulate the distributions of the polar auxin transporters, PIN1, PIN2, and AUX1, we also investigated the impact of CO treatment on basipetal auxin transport in relation to Fe depletion. **Figure 5D** shows the net amount of indole acetic acid (IAA) undergoing basipetal transport in a 1-cm apical segment after various treatments. With and without iron, the auxin over-producing mutant *yuc1*, and CO overproducing mutant *HY1*-OX had the greatest capacity for basipetal auxin transport. In contrast, under –Fe, *hy1, aux1-7, aux1-7/HY1-OX* lines had the lowest transport of auxin among all lines. Compared to *yuc1*, the high transport activity of auxin was impaired in *yuc1/hy1*, indicating that the presence of CO signaling is required for active transport of auxin.

To determine whether auxin contributes to the –Fe response through changes in auxin transport or auxin signal transduction, we examined the responses of the auxin transport mutant, *aux1-7*, and the auxin signaling mutant, *axr1-3*, under Fe depletion. We found that *aux1-7* was more sensitive to –Fe, exhibiting lower FCR activity and basipetal auxin transport than *axr1-3*. *axr1-3* did not differ from wild-type plants during –Fe for FCR activity and basipetal auxin transport (**Figures 5B–D**). Furthermore, *HY1*-OX/*aux1-7* was still sensitive to Fe depletion, indicating over-expression of *HY1* cannot compensate the defects of auxin transport to improve Fe acquisition in the *aux1-7* mutant background (**Figures 5B–D**).

Double mutant analysis identified that *HY1* is epistatic to *YUC1* and *AUX1-7* is epistatic to *HY1*-OX for FCR activity, Fe content, and basipetal auxin transport with or without Fe (**Figures 5B–D**). This suggests, under –Fe, in order to have CO signaling to cause downstream effects (increase in FCR activity, iron content, etc.) the auxin 1–7 transporter is required.

Pharmacological experiments also revealed the importance of auxin and CO in the response to –Fe in *Arabidopsis*. As shown in **Supplementary Figures S6**–**S8**, as compared to untreated plants, the permeable auxin analog NAA significantly improved FCR activity, Fe content, and basipetal auxin transport under –Fe. The addition of CORM2, during –Fe, also increased FCR activity, iron content, and basipetal auxin transport in the wild-type plants but this effect required auxin transporters. The auxin transport inhibitor NPA alone markedly suppressed FCR activity, basipetal auxin transport, and reduced Fe content under –Fe. ZnPPIX treatment, a *HY1* inhibitor, also reduced FCR activity, iron content, and basipetal auxin transport as compared to untreated plants during Fe depletion.

### Under Fe Deficiency, Auxin Effects on NO generation are *HY1* Dependent and Auxin Transporters are needed for NO Generation

**FIGURE 6:**
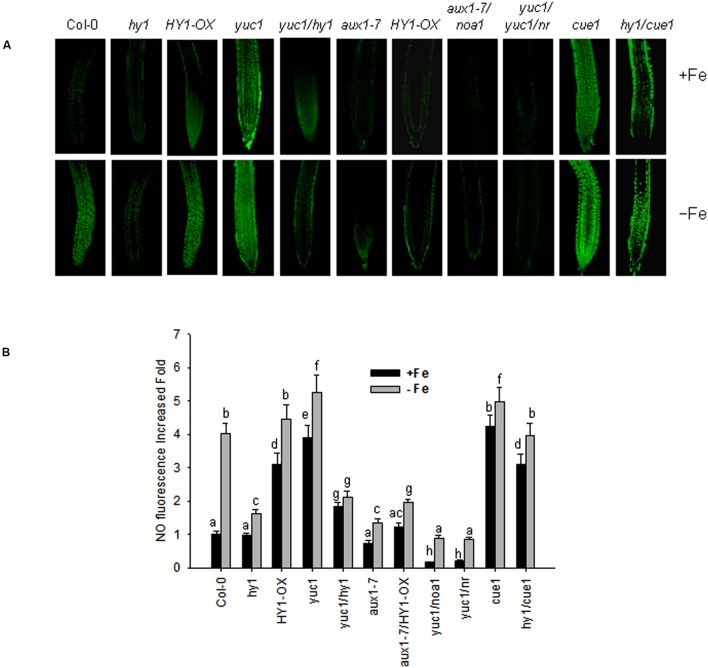
**Iron deficiency stress induced NO generation.** Wild-type Col-0, *hy1, HY1-OX, yuc1, yuc1/hy1, aux1-7, aux1-7/HY1-OX, yuc1/noa1, yuc1/nr, cue1*, and *hy1/cue1* lines were grown on +Fe media and then shifted or not to –Fe media, for 1 day. Green fluorescence indicates NO **(A)**. The corresponding relative fluorescence intensities **(B)** were recorded. Data are the means ± SD (*n* = 15), and bars with different letters are significantly different at *p* < 0.05 (Tukey’s test). BAR = 0.1 cm.

*Arabidopsis noa1* and *nia1/nia2* (termed *nr*) mutants are unable to accumulate sufficient amounts of NO (Guo et al., 2003; Wang et al., 2010), whereas *cue1* produces excess amounts of NO (He et al., 2004). NO was reported to regulate the plant’s response to –Fe (Chen et al., 2010). To unravel the role of cross-talk between CO and NO signaling under –Fe stress in *Arabidopsis*, we examined NO level of the lines subjected to various treatments using the NO dye, DAF-FMDA. With iron, the *yuc1, cue1, HY1*-OX, and *yuc1*/*hy1*, *hy1*/*cue1* lines had higher levels of NO as compared to wild-type (**Figures 6A,B**). The NO levels in *yuc1/hy1* was weaker than in *yuc1*, suggesting that the effects of auxin on the generation of NO are *HY1* dependent. *hy1/cue1* and *cue1* both had higher NO levels than wild-type plants (**Figures 6A,B**). –Fe induced an increase in NO generation, among which there was greatest generation in the yuc1 (auxin over-accumulating) and cue1 (NO over-accumulating) lines. By contrast, NO generation was very low in *hy1* or *yuc1/hy1* under –Fe conditions. In the *yuc1/noa1* and *yuc1/nr* mutants, the NO generation induced by –Fe was lower than in *yuc1*.

**FIGURE 7:**
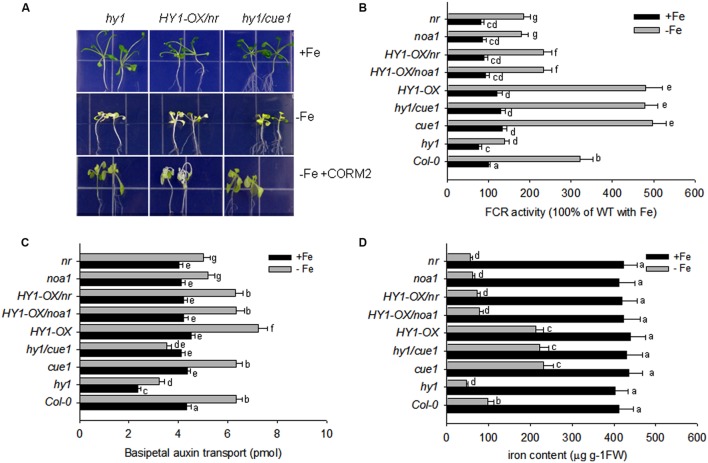
**Auxin and CO modulate the response to –Fe stress in *Arabidopsis*. (A)** The phenotypes of *hy1*, *HY1-OX/nr*, and *hy1/cue1* mutants grown on +Fe, –Fe, or –Fe+CORM2 media for 1 week. All lines were first grown on +Fe media for 2 weeks and then subjected to the specified treatment for 1 week. Lines kept on +Fe media were used as the positive control. **(B–D)** FCR activity, Fe content, and root basipetal auxin transport in the wild-type Col-0, *hy1*, and *HY1-OX* lines upon exposure to –Fe stress. The wild-type Col-0, *hy1*, and *HY1-OX* lines were grown on +Fe media for 2 weeks and then shifted to –Fe media (–Fe), or +Fe media (+Fe) for 1 week before measurements were taken. The FCR activity **(B)**, Fe content **(C)**, and basipetal auxin transport **(D)** were measured after 1 week of –Fe stress. Data are the means ± SD (*n* = 12), and bars with different letters are significantly different at *p* < 0.05 (Tukey’s test).

Accordingly, FCR activity and Fe content were higher in *HY1*-OX and *cue1*, and lower in *hy1*, *noa1*, and *nr* as compared to wild-type plants grown without iron. Furthermore, under –Fe, *hy1/cue1* had higher FCR activity and Fe content than *hy1*. However under –Fe, *HY1*-OX*/nr* had lower FCR activity, Fe content, and basipetal auxin transport than *HY1-OX* (**Figures 7B–D**). Under –Fe, basipetal auxin transport was lower in *hy1* and *hy1/cue1* lines and higher in *HY1*-OX as compared to wild-type (**Figure 7C**). Under –Fe, basipetal auxin transport was significantly less in *noa1*, *nr, hy1, and hy1/cue1 as* compared to wild-type (**Figure 7C**).

Our other experiments also confirmed a role for NO signaling in the response to –Fe in *Arabidopsis*. The treatment with the NO scavenger, cPTIO, greatly suppressed FCR activity induced by Fe depletion, as well as NO generation (**Supplementary Figures S6** and **S9**). Treatments with the NR inhibitor tungstate, or the NO synthase enzyme inhibitor L-NAME, also arrested the increase in FCR activity under –Fe conditions (**Supplementary Figure S6**). By contrast, the treatment with the NO donor (SNAP), increased FCR activity and Fe content under –Fe conditions as compared to untreated plants (**Supplementary Figures S6** and **S8**), However, treatment with the HY1 enzyme inhibitor ZnPPIX, or the auxin transport inhibitor, NPA, reduced NO production, FCR activity, and Fe content as compared to untreated plants (**Supplementary Figure S6**, **S8**, and **S9**). Moreover under –Fe conditions, for the *cue1* mutant, in which NO is constitutively produced, FCR activity and Fe content is increased as compared to wild-type plants (**Figures 7B,D**). Also, the treatments with SNAP, the NO donor, did not have significant effect on the basipetal auxin transport under –Fe status (**Supplementary Figure S7**).

### Auxin, CO, and NO Cooperatively Modulate the –Fe Response

**FIGURE 8:**
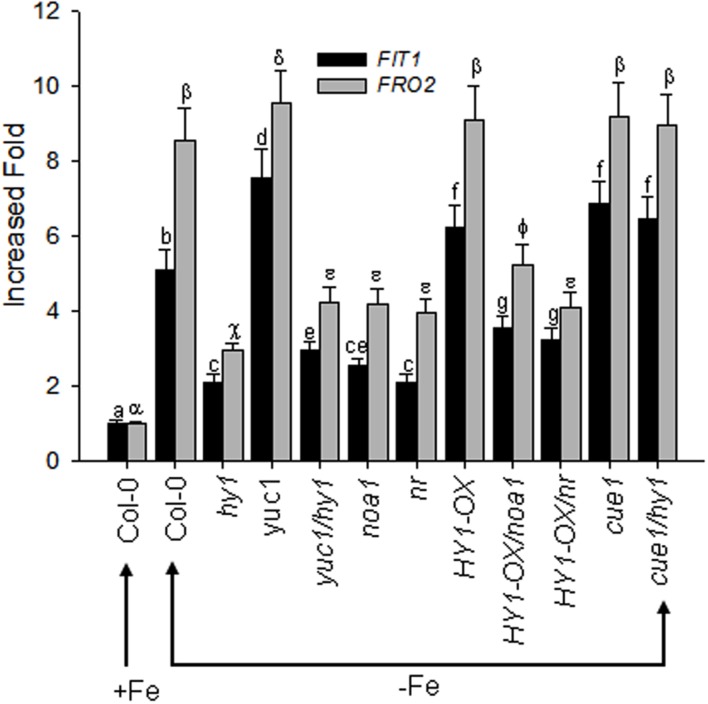
**Differential *FIT1* and *FRO2* expression in CO–, NO–, and auxin-related mutants in response to –Fe stress.** Two-week-old wild-type *Arabidopsis* Col-0 seedlings, CO-related lines (*hy1* and *HY1-OX*), auxin-related mutant (*yuc1*) and NO-related mutants (*cue1, noa1, nr*), and double mutants (*yuc1/hy1, HY1-OX/nr, HY1-OX/noa1, cue1/hy1)* were transferred to –Fe media. The *FIT1* and *FRO2* gene expression in the roots were analyzed by RT-PCR after 24 h of treatment. Tubulin gene expression was used as a control. Data are the means ± SD (*n* = 9), and bars with different letters are significantly different at *p* < 0.05 (Tukey’s test).

The bHLH transcriptional factor FIT1 up-regulates the expression of ferric reduction oxidase 2 (FRO2) in Fe-deficient *Arabidopsis*. We found that Fe depletion caused elevated transcription of *FIT1* and *FRO2* in wild-type plants, and this effect was greater in the *yuc1* line, but less in *hy1, yuc1/hy1, noa1, nr, HY1-OX/noa1, and HY1-OX/nr* (**Figure 8**). Consistent with the observations, as compared to untreated plants, the treatment with either CORM2 (CO donor) or NAA (auxin) enhanced the expression of *FIT1* and *FRO2*, whereas NPA (auxin inhibitor), ZnPPIX (HY1 inhibitor), cPTIO (NO scavenger), L-NAME (NO synthase inhibitor), and tungstate (NR inhibitor) treatments suppressed the transcriptions of *FIT1* and *FRO2* in wild-type plants subjected to –Fe depletion (**Supplementary Figure S10**).

## Discussion

### CO Modulates the –Fe Response in *Arabidopsis*

Numerous studies suggest that CO is a gas messenger that regulates the plant’s response to multiple abiotic stresses (Han et al., 2008; Xuan et al., 2008; Bai et al., 2012; Li et al., 2013). In the present study, we found that CO enhances the tolerance to –Fe in *Arabidopsis*. Heme oxygenase encoded by HY1 is mainly responsible for CO generation in response to various environmental stimuli in *Arabidopsis* (Li et al., 2013). Our experiments showed that inhibiting heme oxygenase activity by using specific inhibitor ZnPPIX reduced the activity of FCR and decreased Fe content as compared to untreated plants (**Supplementary Figures S6** and **S8**), suggesting that CO generation modulates the response to –Fe in *Arabidopsis*. –Fe induces heme oxygenase activity and CO generation in wild-type, but not in *hy1* null mutant (**Figure 1A**). This suggests that *HY1* is needed for increased heme oxygenase activity and CO accumulation for plants. By contrast, *HY1*-OX transgenic line showed higher heme oxygenase activity and CO accumulation than wild-type plants (**Figure 1A**). The *hy1* null mutant was more sensitive to –Fe than the wild-type, exhibiting lower levels of chlorophyll content, FCR activity, and endogenous Fe content. In contrast, the transgenic line over-expressing *HY1* was more tolerant to –Fe than wild-type plants (**Figure 2**). The treatment with artificial CO donor, CORM2, increased chlorophyll content, FCR activity, and Fe content in wild-type and *hy1* lines subjected to –Fe, and treatment with CORM2 up-regulated *FIT1*/*FRO2* expression in wild-type plants (**Figure 2**; **Supplementary Figure S10**). In agreement with previous research (Li et al., 2013), our results indicate that CO improves the ability of *Arabidopsis* to cope with –Fe. In *Arabidopsis*, heme oxygenase encoded by HY1 degrades porphyrin into biliverdin, CO and ionic Fe (Schmitt, 1997). Therefore, induced heme oxygenase activity improves the supply of ionic Fe during short term –Fe. However, this pathway is not sustainable due to the limited amounts of porphyrin in *Arabidopsis*. In fact, elevated CO level through HY1 activity initiates a downstream cascade of events that enhance Fe uptake by increasing the expression of *FIT1* (**Figure 8**). Our finding that the addition of the CO donor CORM2 increases chlorophyll, FCR activity, and Fe content in the *hy1* mutant under Fe depletion (**Figure 2**) also supports this observation.

### CO Promotes Auxin Polar Transport that Improves the Tolerance to –Fe in *Arabidopsis*

Auxin regulates the accumulation of NO that enhances Fe uptake in *Arabidopsis* (Chen et al., 2010). Furthermore, CO and NO interact in *Arabidopsis* to modulate the response to –Fe (Li et al., 2013). We thus reasoned that cross-talk exists between CO and auxin under –Fe. Indeed, we found that auxin induced *HY1* transcription and promoted CO generation (**Figures 3A,B**; **Supplementary Figure S3**) as did –Fe (**Figure 1**). Interestingly, immunoblot analysis revealed two bands of HY1-6HA after auxin or CO biosynthesis inhibitor ZnPPIX treatment in HY1-OX seedlings (**Figure 3C**), which suggests that HY1 undergoes post-transcriptional modification such as protein phosphorylation when subjected to auxin treatment. FIT1 and IRT1, which are involved in the –Fe response, were previously shown to undergo post-transcriptional modification (Barberon et al., 2011; Meiser et al., 2011). Furthermore, the transgenic lines with altered CO accumulation (i.e., *hy1* or *HY1*-OX lines*)* exhibited changes in auxin accumulation in the root tip under Fe depletion, as shown by an increase of *DR5:GFP* fluorescence intensity in *HY1*-OX lines and a decrease in auxin in *hy1* as compared with wild-type and addition of exogenous CO to *hy1* also enhanced auxin-dependent fluorescence (**Figure 4**). These observations suggest that CO increases auxin accumulation in the root tip. PIN1 and PIN2 are both important shoot-to-root auxin transporters (Blilou et al., 2005). A previous study showed that localized treatment with Fe ions altered the distribution of auxin in *Arabidopsis* seedlings (Giehl et al., 2012). We found that more PIN2 accumulated in the *HY1*-OX lines which produced a higher level of CO than wild-type, and addition of exogenous CO increased PIN2 accumulation in the *hy1* mutant impaired in CO production under –Fe (**Figures 4B–D**). This finding correlates with the pattern of auxin distribution in the root tip under –Fe (**Figure 4A**), and suggests that CO promotes auxin redistribution under –Fe. In agreement with the conclusion that CO affects auxin distribution in the root tips by increasing the accumulation of PIN2, we also found that –Fe stress caused an increased number of lateral roots and root hairs in wild-type plants. In contast, the *hy1* line impaired in CO generation did not (**Supplementary Figure S5**). *HY1*-OX, which accumulates a high level of of CO, also exhibited increased lateral root and root hair densities as compared to wild-type plants grown with or without Fe (**Supplementary Figure S5**). Given that plants adjust their lateral root architecture and root hair density to adapt to –Fe stress (Ling et al., 2002; Graziano and Lamattina, 2007), and that CO is an essential signal in root development (Guo et al., 2008), we propose that CO regulates lateral root architecture and root hair density under –Fe stress.

We also evaluated basipetal auxin transport in wild-type, *hy1*, and *HY1*-OX lines, as well as in the auxin over-accumulating line *yuc1* and the auxin transport defective mutant *aux1-7* (**Figure 5D**). Basipetal auxin transport was increased in *HY1*-OX and *yuc1* lines and decreased in *hy1* plants as compared to wild-type plants (**Figure 5D**). As compared to untreated plants, addition of exogenous CO also enhanced basipetal auxin transport (**Supplementary Figure S7**), suggesting that CO generation promotes basipetal auxin transport by affecting the accumulation of PIN1 and PIN2 (**Figure 4**). NPA treatment that impairs auxin transport also caused a dramatic reduction in Fe content and down-regulation of *FIT1* and *FRO2* in *Arabidopsis* subjected to –Fe stress (**Supplementary Figures S8** and **S10**). This suggests that auxin transport is critical in the plants response to –Fe. Suppressing CO generation using the HY1 inhibitor ZnPPIX impaired basipetal auxin transport (**Supplementary Figure S7**) and reduced Fe content under –Fe stress (**Supplementary Figure S8**). Conversely, treatment with the CO donor CORM2 increased basipetal auxin transport and Fe uptake as compared to untreated plants. These results confirm that CO promotes basipetal auxin transport that favors the adaptation of *Arabidopsis* under –Fe stress.

### CO Interacts with Auxin and NO Signals to Regulate the Fe Deficiency Response in *Arabidopsis*

Nitric oxide, another gas messenger, plays multiple regulatory roles in processes such as systemic acquired resistance and the hypersensitive response (Guo et al., 2003; He et al., 2004; Wendehenne et al., 2004; Scheler et al., 2013). Recently, NO has been shown to increase FCR activity in roots and enhances the tolerance to –Fe stress in various plants (Graziano and Lamattina, 2007; Chen et al., 2010). Furthermore, NO acts downstream of auxin signaling (Chen et al., 2010). Our results confirm that the NO signaling is involved in the tolerance of *Arabidopsis* to –Fe stress. *noa1* and *nr*, which are unable to generate NO, show lower levels of Fe content, FCR activity, and basipetal auxin transport under –Fe stress as compared to wild-type plants, while *cue1*, which accumulates high levels of endogenous NO, is tolerant to –Fe stress (**Figure 7**). Similar to the previous findings (Chen et al., 2010), we found that suppressing NO generation using cPTIO, L-NAME, and tungstate caused down-regulation of *FIT1* and *FRO2* (**Supplementary Figure S10**) and a marked reduction of Fe content (**Supplementary Figure S8**). A previous study suggests that the redistribution of auxin triggers NO synthesis and release that initiates the expression of –Fe response genes to enhance Fe uptake (Chen et al., 2010). In agreement with this hypothesis, we found that suppressing auxin transport through NPA treatment or loss of *AUX* (i.e., *aux1-7)* decreased NO release under –Fe stress (**Figure 6**, **Supplementary Figure S9**), while the auxin over-accumulation mutant *yuc1* produced high levels of NO (**Figure 6**). These findings demonstrate the interplay between auxin and NO under –Fe stress again. In the present study, we also unraveled that by manipulating the CO signal, including treatment with ZnPPIX, a specific inhibitor of HY1, knockout of *HY1* (i.e., *hy1*), treatment with the CO donor, CORM2, and overexpressing *HY1* (i.e., *HY1*-OX*)*, polar auxin transport was affected (**Figure 5**; **Supplementary Figure S7**), as evidenced by corresponding PIN1/2 protein accumulation pattern, *DR5:GFP* fluorescence intensity (**Figure 4**), lateral root number and the root hair density (**Supplementary Figure S5**). Ultimately, the NO levels were altered in a corresponding manner (**Figure 6**). Changes in NO levels affected expression of *FIT1*/*FRO2* which directly determines the capacity of Fe acquisition (**Figure 8**). These results suggest that CO effects auxin and NO signals in response to –Fe stress in *Arabidopsis*.

In agreement with our findings, recent studies have suggested that both CO and NO are involved in the plant response to various environmental stresses, including –Fe (Hartsfield, 2002; Guo et al., 2008; Bai et al., 2012; Li et al., 2013). However, the integrative signaling pathway for CO and NO remains largely unknown. Xie et al. (2011) reported that the NOA1/NR-dependent NO signal and HY1-dependent CO signal function in both compensatory and synergistic modes to modulate plant tolerance to salt stress (Xie et al., 2008, 2011). Like *noa1* and *nr*, we found that *hy1* showed increased sensitivity to –Fe stress. However, increasing NO content rescued the sensitivity of *hy1* to –Fe (*hy1cue1* mutant), in agreement with a previous report that NO directly triggers FCR activity and improves Fe uptake (Graziano and Lamattina, 2007; Chen et al., 2010). Furthermore, increasing CO generation did not increase FCR activity or Fe acquisition in *noa1* or *nr* mutants impaired in NO production in our study or a previous study (Bai et al., 2012), suggesting that NO signaling is downstream to CO signaling for FCR activity and Fe content. A recent study has showed that a combination of –Fe and cadmium stress induced NO generation (Han et al., 2008). However, in contrast to our findings, this study found that NO accumulated in *hy1* roots exposed to –Fe stress. Another study found that transgenic *HY1*-OX lines generate more NO than the wild-type after –Fe treatment, while a knock-down of *HY1* expression reduces NO accumulation (Li et al., 2013), which is in agreement with our findings. The discrepancies of NO generation in response to –Fe treatment between these studies may be due to differences in experimental conditions. In the first study, seedlings were grown on solid vertical medium for 5 days and then transferred by hand to +Fe medium for a 3-day treatment. However, young seedlings can easily be wounded when moved by hand, and this may have caused NO to accumulate. As suggested in the second study (Li et al., 2013), we used a liquid-culture system. This system is widely used in Fe stress studies, as it does not damage young seedlings.

In summary, our findings demonstrate that –Fe induces heme oxygenase enzyme-dependent CO generation, which promotes basipetal polar auxin transport in root tip by altering the asymmetric accumulation of PIN1 and PIN2. The elevated level of auxin in the root tip promotes Fe acquisition by up-regulating the expression of Fe-uptake genes *FIT1* and *FRO2*. Our study unravels the interactions among CO, auxin, and NO signals to cope with –Fe in *Arabidopsis*.

## Author Contributions

LY, YG, and JJ performed the experiments, data analysis, and drafted the manuscript. HW, KH-S, EA, and YL assisted in manuscript preparation and revision. XH and YG conceived the project and finalized the manuscript.

## Conflict of Interest Statement

The authors declare that the research was conducted in the absence of any commercial or financial relationships that could be construed as a potential conflict of interest.

## Supplementary material

FIGURE S1 **The HY1-HA expression level in different individual transgenic lines that overexpress HY1-HA.** The proteins from different individual lines that over-express HY1-HA were extracted for determining the HY1-HA protein content by immunoblot analysis using anti-HA antibodies. The total protein was stained with Coomassie blue (ribulose-1,5-bisphosphate carboxylase/oxygenase [Rubisco]) to confirm equal loading.

FIGURE S2 **Time-course analyses of HY1 transcripts under –Fe or +Fe.** Two-week-old wild-type Col-0 lines under –Fe or +Fe medium were used to measure HY1 transcripts at the indicated times. Data are the means ± SD (*n* = 3).

FIGURE S3 **Auxin induced heme oxygenase activity and CO generation.** Two-week-old wild-type Col-0 were treated with 100 nM NAA for indicated time, and the heme oxygenase activity and CO generation were measured. Data are the means ± SD (*n* = 3).

FIGURE S4 **Expression patterns of *AUX1-YFP* in the root tip of wild-type Col-0, *hy1*, *HY1-OX* lines, and *hy1* line treated with CORM2.** The seedlings were treated with 100 nM CORM2 after 12 h of –Fe stress, and YFP fluorescence were recorded.

FIGURE S5 **Carbon monoxide (CO) modulates root hair and lateral root development. (A,B)** Root hair phenotype of Col-0, *hy1*, and *HY1-OX* lines grown in +Fe, –Fe, and –Fe+CORM2 media for 1 week. **(C)** Col-0, *hy1*, and *HY1-OX* lines grown on +Fe, –Fe, or –Fe+CORM2 media for 1 week.

FIGURE S6 **The effects of different inhibitors on FCR activity in the wild-type Col-0 lines with or without –Fe treatment.** Two-week-old wild-type-lines were treated with 100 nM NAA, 10 μM NPA, 200 μM ZnPPIX, 300 μM cPTIO, 1 mM L-NAME, 10 mM tungstate, 100 nM CORM2, 100 nM CORM2+10 μM NPA, 30 μM SNAP, 30 μM GSNO, and 30 μM SNAP+10 μM NPA, respectively, for 1 week, and the FCR activity was measured. Data are the means ± SD (*n* = 12), and bars with different letters are significantly different at *p* < 0.05 (Tukey’s test).

FIGURE S7 **The effects of different inhibitors on auxin transport in the roots of wild-type Col-0 lines with or without –Fe treatment.** Two-week-old wild-type Col-0 lines were treated with 100 nM NAA, 10 μM NPA, 200 μM ZnPPIX, 300 μM cPTIO, 1 mM L-NAME, 10 mM tungstate, 100 nM CORM2, 100 nM CORM2+10 μM NPA, 30 μM SNAP, 30 μM GSNO, and 30 μM SNAP+10 μM NPA, respectively, for 1 week, and basipetal auxin transport was measured. Data are the means ± SD (*n* = 9), and bars with different letters are significantly different at *p* < 0.05 (Tukey’s test).

FIGURE S8 **The effects of different inhibitors on Fe content in roots of wild-type Col-0 lines with or without –Fe treatment.** Two-week-old wild-type Col-0 lines were treated with 100 nM NAA, 10 μM NPA, 200 μM ZnPPIX, 300 μM cPTIO, 1 mM L-NAME, 10 mM tungstate, 100 nM CORM2, 100 nM CORM2+10 μM NPA, 30 μM SNAP, 30 μM GSNO, and 30 μM SNAP+10 μM NPA, respectively, for 1 week, and the Fe content was measured. Data are the means ± SD (*n* = 9), and bars with different letters are significantly different at *p* < 0.05 (Tukey’s test).

FIGURE S9 **The effect of various inhibitors on NO generation after –Fe stress.** Two-week-old wild-type Col-0 lines were treated with 100 nM NAA, 10 mM NPA, 300 μM cPTIO, 1 mM L-NAME, 10 mM tungstate, 200 μM ZnPPIX, 100 nM CORM2, 100 nM CORM2+10 μM NPA, and 30 μM SNAP, respectively, for 1 day, and the green fluorescence **(A)** and the corresponding relative fluorescence intensity **(B)** in the roots were recorded. Data are the means ± SD (*n* = 9), and bars with different letters are significantly different at *p* < 0.05 (Tukey’s test).

FIGURE S10 **The effects of various inhibitors on *FIT1* and *FRO2* expression in the roots of wild-type Col-0 after –Fe stress.** Two-week-old Col-0 seedlings were treated with the indicated inhibitors for 1 day, and *FIT1* **(left)** and *FRO2* **(right)** expression in the root were measured by qRT-PCR analysis. Data are the means ± SD (*n* = 3), and bars with different letters are significantly different at *p* < 0.05 (Tukey’s test).

## Abbreviations

CORM2: tricarbonyldichlororuthenium (II) dimer
cPTIO: 2-4-carboxyphenyl-4, 4, 5, 5- tetra-methylimidazoline- 1- oxyl- 3- oxide
DAF-FMDA: 4-amino-5-methylamino-2,7-di-fluorofluorescein diacetate
–Fe: iron deficiency
GSNO: *S*-Nitrosoglutathione
L-NAME: N^G^-Nitro-L-arginine Methyl Ester
NAA: a-naphthaleneacetic acid
NPA: *N*-1-naphthylphtha- lamic acid
SNAP: *S*- nitroso- *N*- acetylpenicillamine
ZnPPIX: zinc protoporphyrin IX

## References

[B1] Bai X. G. Chen J. H. Kong X. X. Todd C. D. Yang Y. P. Hu X. Y. (2012). Carbon monoxide enhances the chilling tolerance of recalcitrant *Baccaurea ramiflora* seeds via nitric oxide-mediated glutathione homeostasis. *Free Radic. Biol. Med.* 53 710–720. 10.1016/j.freeradbiomed.2012.05.042 22683602

[B2] Barberon M. Zelazny E. Robert S. Conejero G. Curie C. Friml J. (2011). Monoubiquitin-dependent endocytosis of the iron-regulated transporter 1 (IRT1) transporter controls iron uptake in plants. *Proc. Natl. Acad. Sci. U.S.A.* 108 E450–E458. 10.1073/pnas.1100659108 21628566 PMC3156158

[B3] Besson-Bard A. Pugin A. Wendehenne D. (2008). New insights into nitric oxide signaling in plants. *Annu. Rev. Plant Biol.* 59 21–39. 10.1146/annurev.arplant.59.032607.092830 18031216

[B4] Blilou I. Xu J. Wildwater M. Willemsen V. Paponov I. Friml J. (2005). The PIN auxin eﬄux facilitator network controls growth and patterning in *Arabidopsis* roots. *Nature* 433 39–44. 10.1038/nature03184 15635403

[B5] Briat J. F. Fobis-Loisy I. Grignon N. Lobreaux S. Pascal N. Savino G. (1995). Cellular and molecular aspects of iron metabolism in plants. *Biol. Cell* 84 69–81. 10.1016/0248-4900(96)81320-7

[B6] Chen W. W. Yang J. L. Qin C. Jin C. W. Mo J. H. Ye T. (2010). Nitric oxide acts downstream of auxin to trigger root ferric-chelate reductase activity in response to iron deficiency in *Arabidopsis.* *Plant Physiol.* 154 810–819. 10.1104/pp.110.161109 20699398 PMC2948983

[B7] Clough S. J. Bent A. F. (1998). Floral dip: a simplified method for Agrobacterium-mediated transformation of *Arabidopsis thaliana*. *Plant J.* 16 735–743. 10.1046/j.1365-313x.1998.00343.x 10069079

[B8] Curie C. Briat J. F. (2003). Iron transport and signaling in plants. *Annu. Rev. Plant Biol.* 54 183–206. 10.1146/annurev.arplant.54.031902.135018 14509968

[B9] Davis S. J. Kurepa J. Vierstra R. D. (1999). The *Arabidopsis thaliana* HY1 locus, required for phytochrome-chromophore biosynthesis, encodes a protein related to heme oxygenases. *Proc. Natl. Acad. Sci. U.S.A.* 96 6541–6546. 10.1073/pnas.96.11.6541 10339624 PMC26918

[B10] Giehl R. F. Lima J. E. Von Wiren N. (2012). Localized iron supply triggers lateral root elongation in *Arabidopsis* by altering the AUX1-mediated auxin distribution. *Plant Cell* 24 33–49. 10.1105/tpc.111.092973 22234997 PMC3289578

[B11] Graziano M. Lamattina L. (2007). Nitric oxide accumulation is required for molecular and physiological responses to iron deficiency in tomato roots. *Plant J.* 52 949–960. 10.1111/j.1365-313X.2007.03283.x 17892445

[B12] Green L. S. Rogers E. E. (2004). FRD3 controls iron localization in *Arabidopsis*. *Plant Physiol.* 136 2523–2531. 10.1104/pp.104.045633 15310833 PMC523319

[B13] Guo F. Q. Crawford N. M. (2005). Arabidopsis nitric oxide synthase1 is targeted to mitochondria and protects against oxidative damage and dark-induced senescence. *Plant Cell* 17 3436–3450. 10.1105/tpc.105.037770 16272429 PMC1315380

[B14] Guo F. Q. Okamoto M. Crawford N. M. (2003). Identification of a plant nitric oxide synthase gene involved in hormonal signaling. *Science* 302 100–103. 10.1126/science.1086770 14526079

[B15] Guo K. Xia K. Yang Z. M. (2008). Regulation of tomato lateral root development by carbon monoxide and involvement in auxin and nitric oxide. *J. Exp. Bot.* 59 3443–3452. 10.1093/jxb/ern194 18653694 PMC2529230

[B16] Han Y. Zhang J. Chen X. Gao Z. Xuan W. Xu S. (2008). Carbon monoxide alleviates cadmium-induced oxidative damage by modulating glutathione metabolism in the roots of *Medicago sativa*. *New Phytol.* 177 155–166. 18028301 10.1111/j.1469-8137.2007.02251.x

[B17] Hartsfield C. L. (2002). Cross talk between carbon monoxide and nitric oxide. *Antioxid. Redox. Signal.* 4 301–307. 10.1089/152308602753666352 12006181

[B18] He Y. Tang R. H. Hao Y. Stevens R. D. Cook C. W. Ahn S. M. (2004). Nitric oxide represses the *Arabidopsis floral* transition. *Science* 305 1968–1971. 10.1126/science.1098837 15448272

[B19] Imsande J. (1998). Iron, sulfur, and chlorophyll deficiencies: a need for an integrative approach in plant physiology. *Physiol. Plant.* 103 139–144. 10.1034/j.1399-3054.1998.1030117.x

[B20] Le Jean M. Schikora A. Mari S. Briat J. F. Curie C. (2005). A loss-of-function mutation in AtYSL1 reveals its role in iron and nicotianamine seed loading. *Plant J.* 44 769–782. 10.1111/j.1365-313X.2005.02569.x 16297069

[B21] Li H. Song J. B. Zhao W. T. Yang Z. M. (2013). AtHO1 is involved in iron homeostasis in an NO-dependent manner. *Plant Cell Physiol.* 54 1105–1117. 10.1093/pcp/pct063 23620481

[B22] Lincoln C. Britton J. H. Estelle M. (1990). Growth and development of the axr1 mutants of *Arabidopsis*. *Plant Cell* 2 1071–1080. 10.2307/3869260 1983791 PMC159955

[B23] Ling H. Q. Bauer P. Bereczky Z. Keller B. Ganal M. (2002). The tomato fer gene encoding a bHLH protein controls iron-uptake responses in roots. *Proc. Natl. Acad. Sci. U.S.A.* 99 13938–13943. 10.1073/pnas.212448699 12370409 PMC129801

[B24] Lingam S. Mohrbacher J. Brumbarova T. Potuschak T. Fink-Straube C. Blondet E. (2011). Interaction between the bHLH transcription factor FIT and ETHYLENE INSENSITIVE3/ETHYLENE INSENSITIVE3-LIKE1 reveals molecular linkage between the regulation of iron acquisition and ethylene signaling in *Arabidopsis*. *Plant Cell* 23 1815–1829. 10.1105/tpc.111.084715 21586684 PMC3123957

[B25] Long T. A. Tsukagoshi H. Busch W. Lahner B. Salt D. E. Benfey P. N. (2010). The bHLH transcription factor POPEYE regulates response to iron deficiency in *Arabidopsis* roots. *Plant Cell* 22 2219–2236. 10.1105/tpc.110.074096 20675571 PMC2929094

[B26] Marschner P. (2011). *Marschner’s Mineral Nutrition of Higher Plants*. San Diego CA: Academic Press.

[B27] Meiser J. Lingam S. Bauer P. (2011). Posttranslational regulation of the iron deficiency basic helix-loop-helix transcription factor FIT is affected by iron and nitric oxide. *Plant Physiol.* 157 2154–2166. 10.1104/pp.111.183285 21972265 PMC3327203

[B28] Müller B. Sheen J. (2008). Cytokinin and auxin interaction in root stem-cell specification during early embryogenesis. *Nature* 453 1094–1097. 10.1038/nature06943 18463635 PMC2601652

[B29] Ottenschläger I. Wolff P. Wolverton C. Bhalerao R. P. Sandberg G. Ishikawa H. (2003). Gravity-regulated differential auxin transport from columella to lateral root cap cells. *Proc. Natl. Acad. Sci. U.S.A.* 100 2987–2991. 10.1073/pnas.0437936100 12594336 PMC151453

[B30] Otterbein L. E. Bach F. H. Alam J. Soares M. Lu H. Wysk M. (2000). Carbon monoxide has anti-inflammatory effects involving the mitogen-activated protein kinase pathway. *Nat. Med.* 6 422–428. 10.1038/74680 10742149

[B31] Pickett F. B. Wilson A. K. Estelle M. (1990). The aux1 mutation of *Arabidopsis* confers both auxin and ethylene resistance. *Plant Physiol.* 94 1462–1466. 10.1104/pp.94.3.1462 16667854 PMC1077399

[B32] Scheler C. Durner J. Astier J. (2013). Nitric oxide and reactive oxygen species in plant biotic interactions. *Curr. Opin. Plant Biol.* 16 534–539. 10.1016/j.pbi.2013.06.020 23880111

[B33] Schmitt M. P. (1997). Utilization of host iron sources by Corynebacterium diphtheriae: identification of a gene whose product is homologous to eukaryotic heme oxygenases and is required for acquisition of iron from heme and hemoglobin. *J. Bacteriol.* 179 838–845. 9006041 10.1128/jb.179.3.838-845.1997 PMC178768

[B34] Scholl R. L. May S. T. Ware D. H. (2000). Seed and molecular resources for *Arabidopsis*. *Plant Physiol.* 124 1477–1480. 10.1104/pp.124.4.1477 11115863 PMC1539300

[B35] Sivitz A. B. Hermand V. Curie C. Vert G. (2012). Arabidopsis bHLH100 and bHLH101 control iron homeostasis via a FIT-independent pathway. *PLoS ONE* 7:e44843 10.1371/journal.pone.0044843 PMC3439455 22984573

[B36] Ulmasov T. Murfett J. Hagen G. Guilfoyle T. J. (1997). Aux/IAA proteins repress expression of reporter genes containing natural and highly active synthetic auxin response elements. *Plant Cell* 9 1963–1971. 10.1105/tpc.9.11.1963 9401121 PMC157050

[B37] Vert G. Grotz N. Dedaldechamp F. Gaymard F. Guerinot M. L. Briat J. F. (2002). IRT1, an *Arabidopsis* transporter essential for iron uptake from the soil and for plant growth. *Plant Cell* 14 1223–1233. 10.1105/tpc.001388 12084823 PMC150776

[B38] Wang H. Y. Klatte M. Jakoby M. Baumlein H. Weisshaar B. Bauer P. (2007). Iron deficiency-mediated stress regulation of four subgroup Ib BHLH genes in *Arabidopsis thaliana*. *Planta* 226 897–908. 10.1007/s00425-007-0535-x 17516080

[B39] Wang P. Du Y. Li Y. Ren D. Song C. P. (2010). Hydrogen peroxide-mediated activation of MAP kinase 6 modulates nitric oxide biosynthesis and signal transduction in *Arabidopsis. Plant Cell* 22 2981–2998. 10.1105/tpc.109.072959 20870959 PMC2965546

[B40] Wendehenne D. Durner J. Klessig D. F. (2004). Nitric oxide: a new player in plant signalling and defence responses. *Curr. Opin. Plant Biol.* 7 449–455. 10.1016/j.pbi.2004.04.002 15231269

[B41] Xie Y. Ling T. Han Y. Liu K. Zheng Q. Huang L. (2008). Carbon monoxide enhances salt tolerance by nitric oxide-mediated maintenance of ion homeostasis and up-regulation of antioxidant defence in wheat seedling roots. *Plant. Cell. Environ.* 31 1864–1881. 10.1111/j.1365-3040.2008.01888.x 18811735

[B42] Xie Y. J. Xu S. Han B. Wu M. Z. Yuan X. X. Han Y. (2011). Evidence of *Arabidopsis* salt acclimation induced by up-regulation of HY1 and the regulatory role of RbohD-derived reactive oxygen species synthesis. *Plant J.* 66 280–292. 10.1111/j.1365-313X.2011.04488.x 21205037

[B43] Xuan W. Zhu F. Y. Xu S. Huang B. K. Ling T. F. Qi J. Y. (2008). The heme oxygenase/carbon monoxide system is involved in the auxin-induced cucumber adventitious rooting process. *Plant Physiol.* 148 881–893. 10.1104/pp.108.125567 18689445 PMC2556841

[B44] Yang L. Fountain J. C. Wang H. Ni X. Ji P. Lee R. D. (2015). Stress sensitivity is associated with differential accumulation of reactive oxygen and nitrogen species in maize genotypes with contrasting levels of drought tolerance. *Int. J. Mol. Sci.* 16 24791–24819. 10.3390/ijms161024791 26492235 PMC4632777

[B45] Yang L. Tian D. Todd C. D. Luo Y. Hu X. (2013). Comparative proteome analyses reveal that nitric oxide is an important signal molecule in the response of rice to aluminum toxicity. *J. Proteome Res.* 12 1316–1330. 10.1021/pr300971n 23327584

[B46] Yuan Y. Wu H. Wang N. Li J. Zhao W. Du J. (2008). FIT interacts with AtbHLH38 and AtbHLH39 in regulating iron uptake gene expression for iron homeostasis in *Arabidopsis*. *Cell Res.* 18 385–397. 10.1038/cr.2008.26 18268542

[B47] Zhao Y. Christensen S. K. Fankhauser C. Cashman J. R. Cohen J. D. Weigel D. (2001). A role for flavin monooxygenase-like enzymes in auxin biosynthesis. *Science* 291 306–309. 10.1126/science.291.5502.306 11209081

