# Supplementary figures and images for: Carbon Monoxide Interacts with Auxin and Nitric Oxide to Cope with Iron Deficiency in Arabidopsis
Source: Front Plant Sci. 2016 Mar 7;7:112. doi: 10.3389/fpls.2016.00112 (PMC4780267; doi:10.3389/fpls.2016.00112)

Figure S1

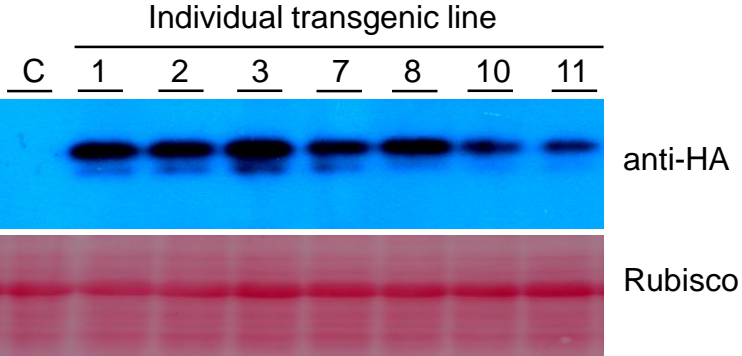

Figure S2

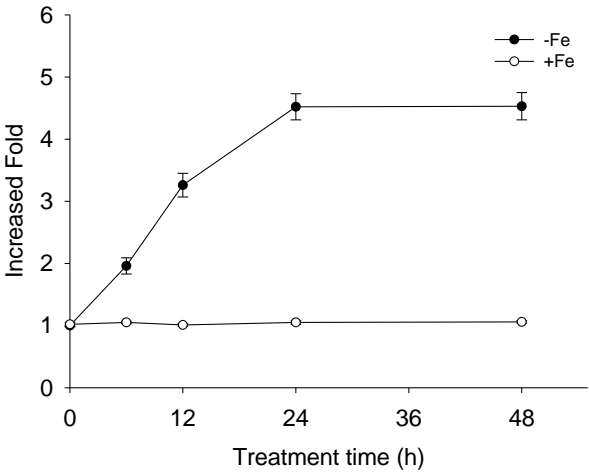

Figure S3

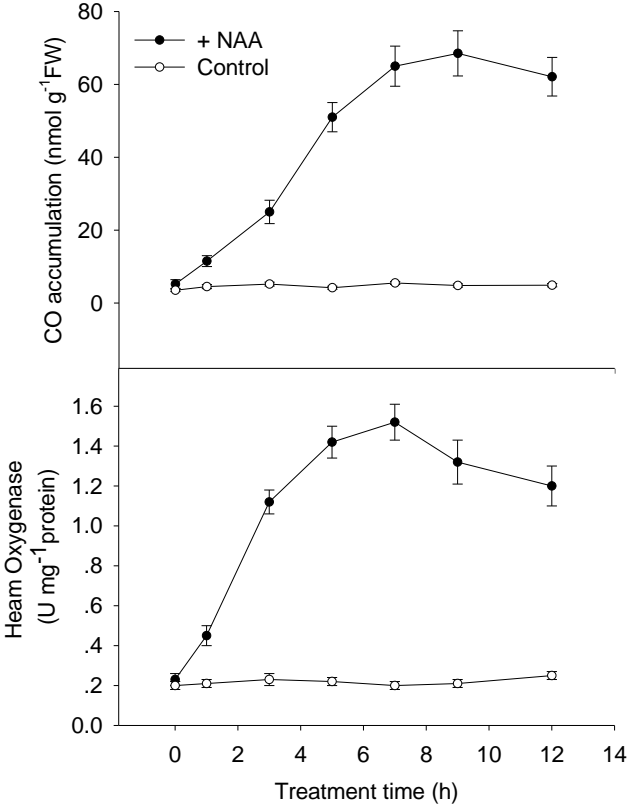

Figure S4

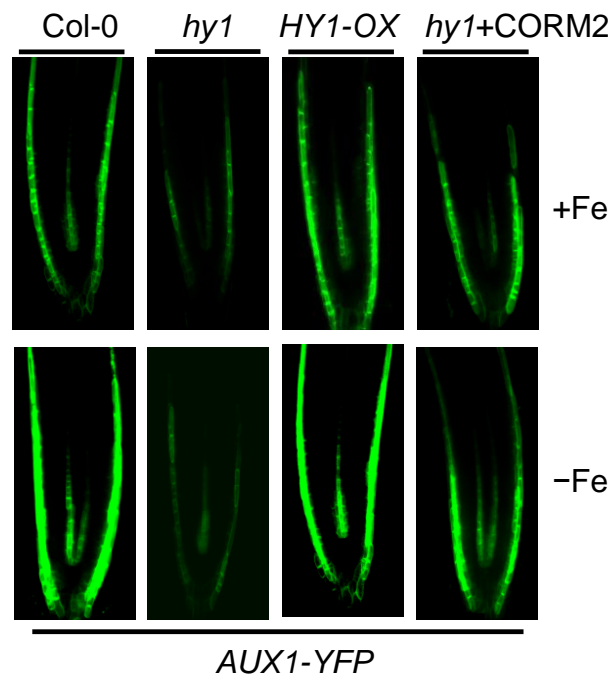

Figure S5

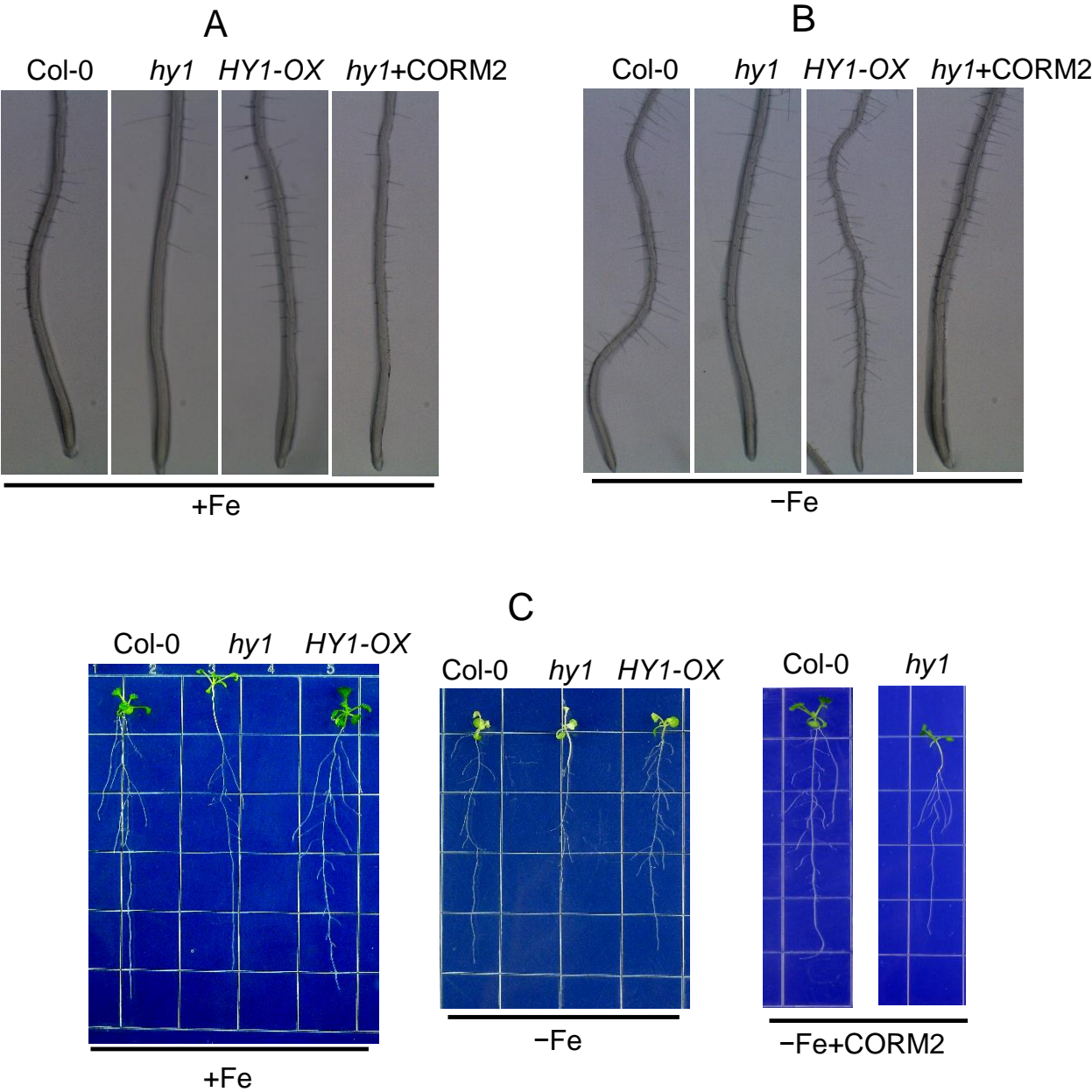

Figure S6

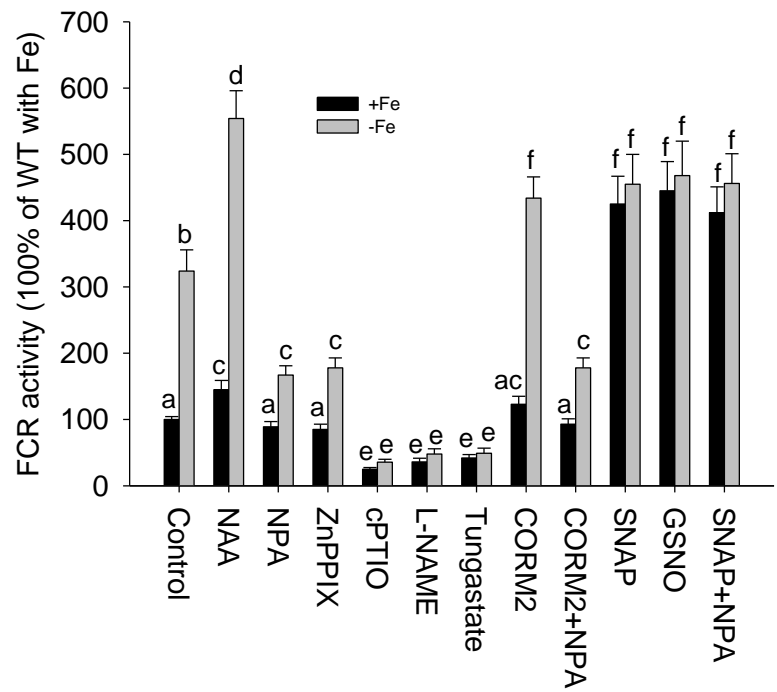

Figure S7

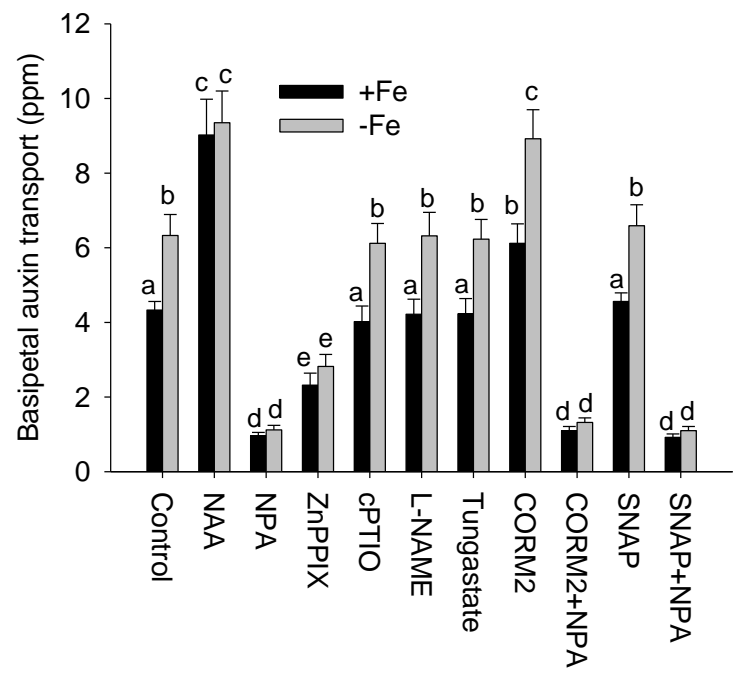

Figure S8

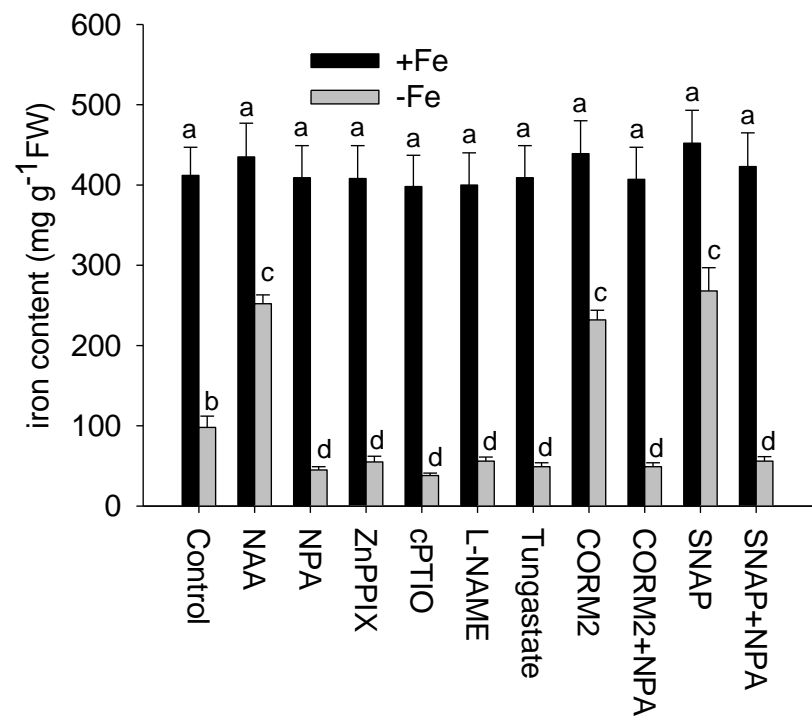

Figure S9

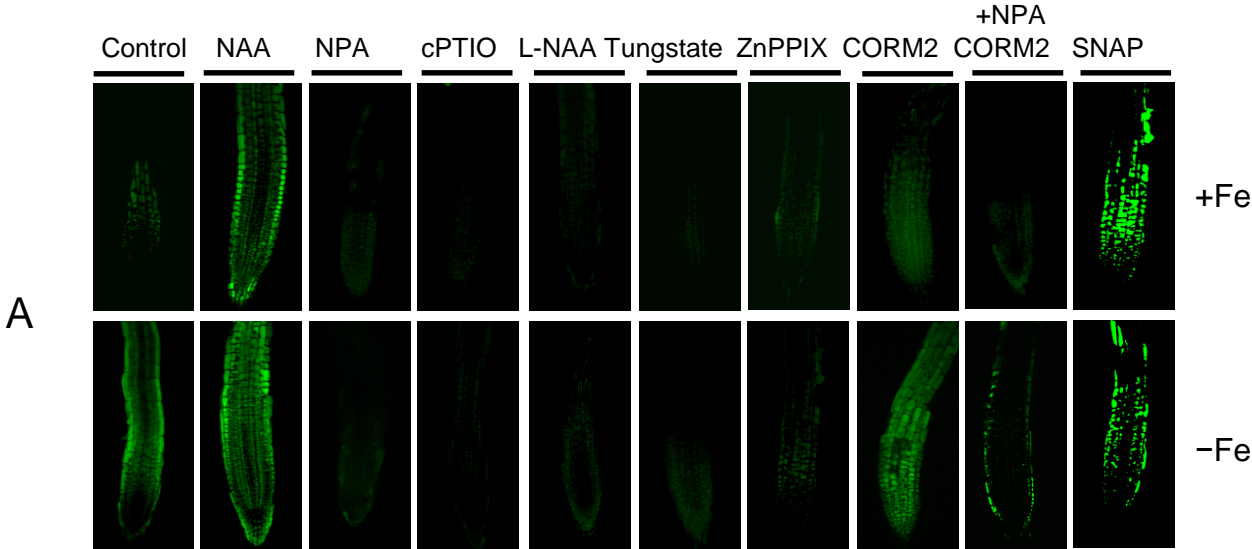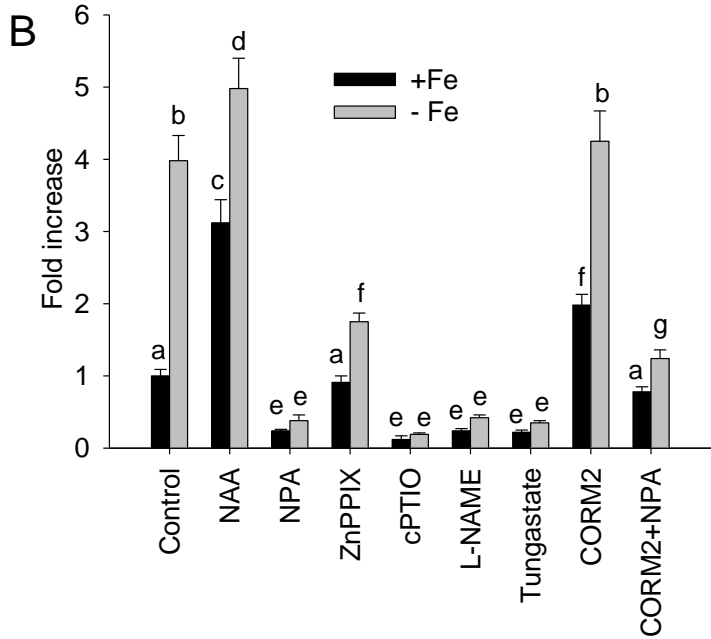

Figure S10

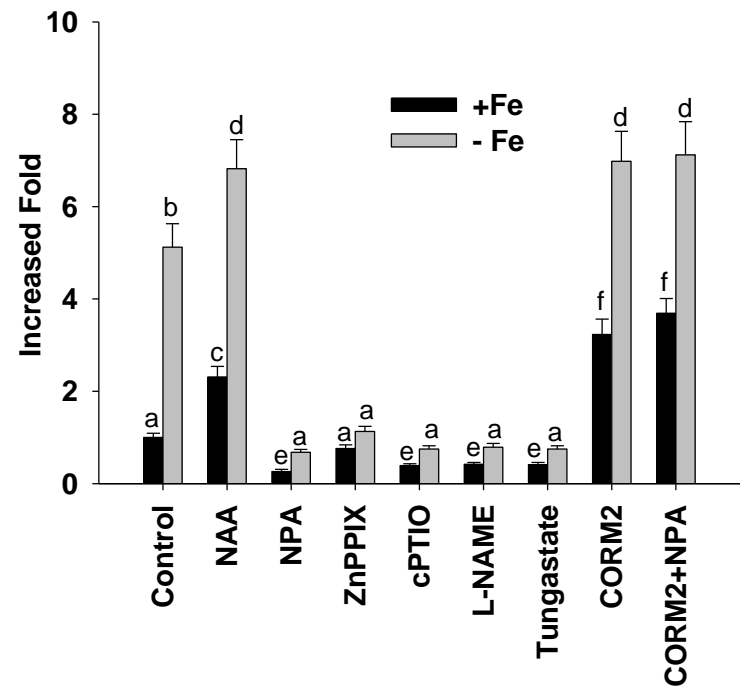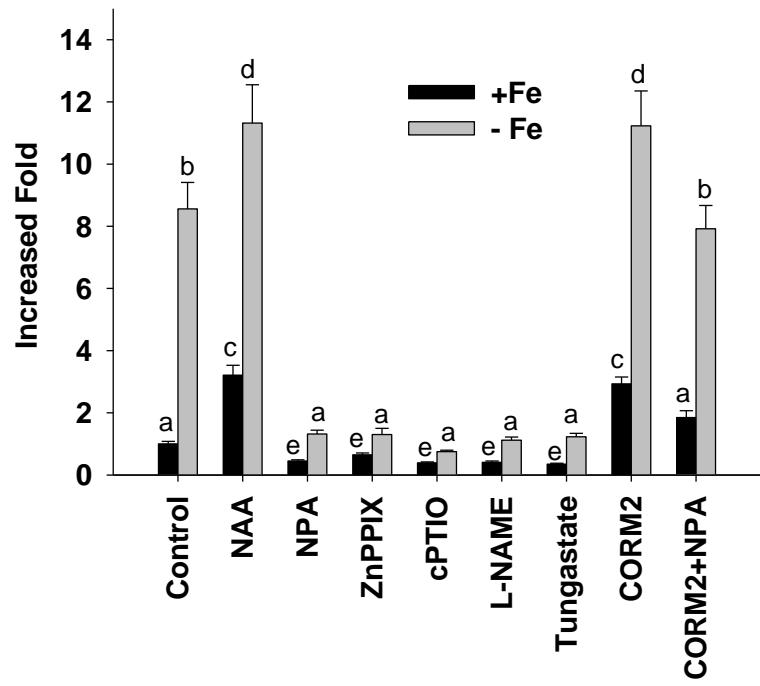

Supplement: FIGURE S1 — The HY1-HA expression level in different individual transgenic lines that overexpress HY1-HA. The proteins from different individual lines that over-express HY1-HA were extracted for determining the HY1-HA protein content by immunoblot analysis using anti-HA antibodies. The total protein was stained with Coomassie blue (ribulose-1,5-bisphosphate carboxylase/oxygenase [Rubisco]) to confirm equal loading. [file Image_1.PDF]
