# Supplementary material for: Carbon Monoxide Interacts with Auxin and Nitric Oxide to Cope with Iron Deficiency in Arabidopsis
Source: Front Plant Sci. 2016 Mar 7;7:112. doi: 10.3389/fpls.2016.00112 (PMC4780267; doi:10.3389/fpls.2016.00112)
Supplement: Supplementary file 2 [file Table_1.DOC]

**Supplementary Table 1**. The prime sequences used in this study.

| **Prime sequences for construct** | |
| --- | --- |
| **pOE-HY1 F(EcoRI)** | **gggccc GAATTC GCGTATTTAGCTCCGATTTCT** |
| **pOE-HY1 R(Hind III)** | **gggccc AAGCTT TCAGGACAATATGAGACGA** |
| **pHY1:GUS-F(KpnI)** | **cggGGTACCccg**ATGGCGTATTTAGCTCCG |
| **pHY1:GUS-R(EcoRI)** | ccgGAATTCcgg GGACAATATGAGACGAAGT |

| **Prime sequences for T-DNA insertion line verification** | |
| --- | --- |
| **cs236-F (for PCR product to sequencing)** | ACTGAGTTTGCTTATGTATTGGA |
| **cs236-R(for PCR product to sequencing)** | TGTAGAAGTGACAAATGAATGCT |
| **Cue1-F** | TCTCGTTCTGATGGCTCCTGTG |
| **Cue1-R** | GTGTAACCGGTGATACTCTCGCC |
| **YUC1-F** | TCCTCGGATTCCATTGCCCAGC |
| **YUC1-R** | ACGAGAGTGTCGTGCTCCACCA |
| **nia1 salk_004164-F** | TAATCTACATAATTGCCCCGC |
| **nia1 salk_004164-R** | CGTTAAACGTTTGCTTCGAAG |
| **nia2 salk_088070-F** | CTTTGGTAGACGCCGAACTC |
| **nia2 salk_088070-R** | TTGGCTTTATCCGAGTGAATG |
| **ATnoa1-F** | TTTCCTCATGGTCACTTCCAC |
| **ATnoa1-R** | TAACCGTCTCCAATCAACCAG |
| **LB** | ATTTTGCCGATTTCGGAAC |

| **Prime sequences for qRT-PCR** | |
| --- | --- |
| **FIT1-F** | GTATCAATCCTCCTGCTT |
| **FIT1-R** | TCTCGGTTACATCATCACT |
| **FRO2-F** | GTAAACAGGTCCAAAACG |
| **FRO2-R** | GTAAAGCACACAAAGATAGG |
| **HY1-F** | TTGCTCATAGTGCTGGTGGA |
| **HY1-R** | TAGTCCACTCCTCTGCAACC |
| **Beta-tubulin-F** | AAGTTCTGGGAAGTGGTT |
| **Beta-tubulin-R** | CTCCCAATGAGTGACAAA |
